# Neural Tissue‐Like, not Supraphysiological, Electrical Conductivity Stimulates Neuronal Lineage Specification through Calcium Signaling and Epigenetic Modification

**DOI:** 10.1002/advs.202400586

**Published:** 2024-07-10

**Authors:** Yu‐Meng Li, Yunseong Ji, Yu‐Xuan Meng, Yu‐Jin Kim, Hwalim Lee, Amal George Kurian, Jeong‐Hui Park, Ji‐Young Yoon, Jonathan C. Knowles, Yunkyu Choi, Yoon‐Sik Kim, Bo‐Eun Yoon, Rajendra K. Singh, Hae‐Hyoung Lee, Hae‐Won Kim, Jung‐Hwan Lee

**Affiliations:** ^1^ Institute of Tissue Regeneration Engineering (ITREN) Dankook University Cheonan Chungcheongnam‐do 31116 Republic of Korea; ^2^ Department of Nanobiomedical Science and BK21 Four NBM Global Research Center for Regenerative Medicine Dankook University Cheonan Chungcheongnam‐do 31116 Republic of Korea; ^3^ Fuel Cell Laboratory Korea Institute of Energy Research (KIER) Daejeon 34129 Republic of Korea; ^4^ Department of Biomaterials Science College of Dentistry Dankook University Cheonan Chungcheongnam‐do 31116 Republic of Korea; ^5^ UCL Eastman‐Korea Dental Medicine Innovation Centre Dankook University Cheonan Chungcheongnam‐do 31116 Republic of Korea; ^6^ Division of Biomaterials and Tissue Engineering UCL Eastman Dental Institute Royal Free Hospital Rowland Hill Street London NW3 2PF UK; ^7^ Department of Chemical and Biomolecular Engineering Yonsei University Seoul 03722 Republic of Korea; ^8^ Mechanobiology Dental Medicine Research Center Dankook University Cheonan Chungcheongnam‐do 31116 Republic of Korea; ^9^ Department of Molecular Biology Dankook University Cheonan 31116 Republic of Korea; ^10^ Cell & Matter Institute Dankook University Cheonan 31116 Republic of Korea; ^11^ Department of Regenerative Dental Medicine College of Dentistry Dankook University Cheonan Chungcheongnam‐do 31116 Republic of Korea

**Keywords:** calcium signaling, electrical conductivity, epigenetic change, neural lineage specification

## Abstract

Electrical conductivity is a pivotal biophysical factor for neural interfaces, though optimal values remain controversial due to challenges isolating this cue. To address this issue, conductive substrates made of carbon nanotubes and graphene oxide nanoribbons, exhibiting a spectrum of conductivities from 0.02 to 3.2 S m^−1^, while controlling other surface properties is designed. The focus is to ascertain whether varying conductivity in isolation has any discernable impact on neural lineage specification. Remarkably, neural‐tissue‐like low conductivity (0.02–0.1 S m^−1^) prompted neural stem/progenitor cells to exhibit a greater propensity toward neuronal lineage specification (neurons and oligodendrocytes, not astrocytes) compared to high supraphysiological conductivity (3.2 S m^−1^). High conductivity instigated the apoptotic process, characterized by increased apoptotic fraction and decreased neurogenic morphological features, primarily due to calcium overload. Conversely, cells exposed to physiological conductivity displayed epigenetic changes, specifically increased chromatin openness with H3acetylation (H3ac) and neurogenic‐transcription‐factor activation, along with a more balanced intracellular calcium response. The pharmacological inhibition of H3ac further supported the idea that such epigenetic changes might play a key role in driving neuronal specification in response to neural‐tissue‐like, not supraphysiological, conductive cues. These findings underscore the necessity of optimal conductivity when designing neural interfaces and scaffolds to stimulate neuronal differentiation and facilitate the repair process.

## Graphical abstract

Neural tissue‐like electrical conductivity (0.02–0.1 S m^−1^) enhances neuronal lineage specification of neural stem/progenitor cells compared to supraphysiological conductivity (3.2 S m^−1^), while other surface properties remain constant. This neurogenic effect is mediated by balanced intracellular calcium dynamics and increased H3 acetylation, highlighting the importance of optimizing conductivity in neural interface design to stimulate neuronal differentiation and facilitate repair.

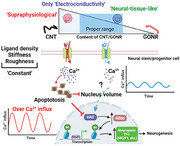

## 1 Introduction

During the tissue regeneration process, adult stem cells migrate away from their specific niche, subsequently engraft and differentiate, sensing the extracellular matrix (ECM).^[^ ^1^, ^2^ ^]^ Among the various physicochemical properties of the ECM influencing stem cell behaviors, electrical conductivity stands out as a significant biophysical cue.^[^ ^3^ ^]^ Native tissues, including nerves, exhibit electrical conductivity ranging up to ≈ 0.6 S m^−1^, depending on tissue type, anatomical location, and directionality.^[^ ^4^, ^5^ ^]^ Neural stem cells demonstrate an ability to adapt to electrical conductivity, differentiating into various neural lineages, including neurons, astrocytes, and oligodendrocytes.^[^ ^6^ ^]^ While mechanisms governing the perception of electrical conductivity remain largely unexplored, some ion channels localized at the plasma membrane are considered the initial responders.^[^ ^7^ ^]^

To accelerate nerve regeneration through stimulation of stem cells, a range of electroconductive substrates have thus been developed, including conductive biopolymers, carbon‐based materials, and metals. It has been largely observed that higher electroconductivity can enhance the neuronal specification of stem cells and neural regeneration.^[^ ^8^, ^9^, ^10^, ^11^ ^]^ However, the specific impact of electrical conductivity remains intertwined with other substrate parameters, like surface topology/roughness, material composition, and cell‐binding ligands. Consequently, a detailed examination of the direct effects of matrix electrical conductivity remains pending.^[^ ^12^, ^13^ ^]^

Cells sense and integrate biophysical cues from ECM to convert them into biochemical signals, including ion influx, activation of a signaling pathway, and transcriptional regulation, which ultimately govern gene expression and cell fate decisions.^[^ ^14^, ^15^, ^16^ ^]^ Within this cell‐ECM signaling process, not only cytoskeletal components but the nuclear elements, such as chromatins,^[^ ^17^ ^]^ are modified, ultimately influencing the expression of genes and their fate like apoptosis and lineage specification.^[^ ^18^ ^]^ This phenomenon has been observed across various cell types, including epithelial cells, fibroblasts, and mesenchymal stem cells.^[^ ^18^, ^19^, ^20^, ^21^, ^22^, ^23^, ^24^, ^25^, ^26^ ^]^ In the context of neural stem cells, nuclear chromatin modification has also been recognized as a significant phenomenon in both the development and the regeneration of neural tissues, such as the brain, spinal cord, and peripheral nerves.^[^ ^27^, ^28^, ^29^ ^]^ However, the question of whether electrical conductivity can lead to chromatin modification and how this phenomenon is linked to neural regeneration remains unanswered.

To this end, we aim to examine the specific impact of electrical conductivity on neural stem/progenitor cell (NSPC) lineage specification and functionality, by using substrates made of carbon nanotube (CNT) and graphene oxide nanofiber. This combination of carbon‐based materials offers a controlled range of conductivity, ranging from 0.02 to 3.2 S m^−1^, covering both “physiological” and “supraphysiological” conductivity levels. Importantly, we can isolate this conductivity from other decisive physicochemical variables, such as surface roughness, cell‐binding ligands, and compositional elements.^[^ ^30^ ^]^ Interestingly, we found that physiological conductivity (0.02–0.1 S m^−1^) promoted neuronal differentiation of NSPCs to a greater extent than supraphysiological conductivity (3.2 S m^−1^). We further identified that supraphysiological conductivity induced an apoptotic process driven by hyperactive intracellular calcium dynamics, whereas physiological conductivity resulted in a balanced intracellular calcium response. Of note, upon physiologically conductive substrates, cells displayed open chromatin structures characterized by H3acetylation (H3ac) and neurogenic transcription factor activation. Our findings highlight the significance of employing conductivity levels that mirror physiological conditions when designing neural interfaces and scaffolds to stimulate neuronal differentiation and facilitate the neural repair process.

## 2 Results and Discussion

### 2.1 Control Over Electrical Conductivity (Physiological versus Supraphysiological) Together with Decoupled Physicochemical Properties

**Figure 1:**
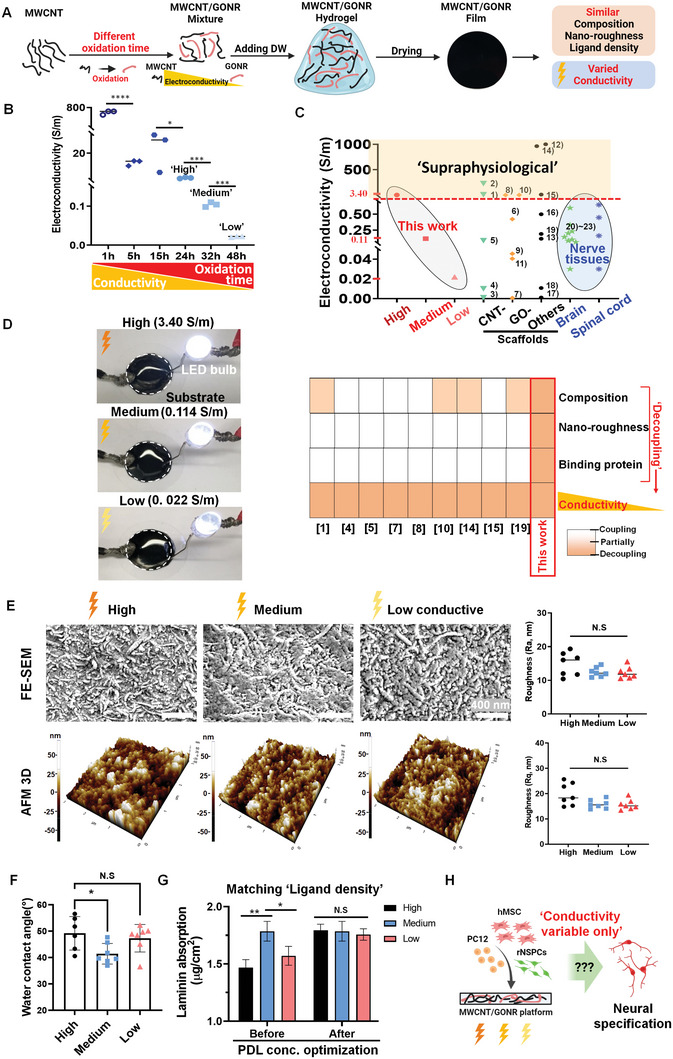
The physiochemical characterization of various electroconductive carbon substrates for investigating neural lineage specification depending solely on the electrical conductivity. A) Schematic illustration of fabrication procedure via different oxidation of MWCNT. B) The electroconductive properties of the carbon films under different oxidation times. C) The electrical conductivity of this work compared to other publications and its unique advantages (Same component, similar nano‐roughness, ECM protein absorption amount) were portrayed, decoupling other key influential parameters to study the electrical conductivity effects solely. The detailed electrical conductivity values of the electroconductive scaffolds from the references are listed in Table S1 (Supporting Information). D) The electrical conductivity was examined by LED light visualization and the four‐probe detection method. A white dot circle from each picture, indicates a substrate with different electroconductivity. E) The surface morphology and roughness characterization by FE‐SEM and AFM, reveals similar topology and roughness. F) The hydrophilicity by the water contacts angle analysis. G) Optimization of laminin‐binding amount for different electroconductive carbon films. H) Schematic illustration demonstrated the motivation point of this study. *P < 0.05, **P < 0.01, ***P < 0.001, ****P < 0.0001 (ANOVA and Tukey posthoc test after confirming normality and distribution symmetry by Shapiro‐Wilk test at a level of 0.05). N.S. indicated there was no significant difference between groups. The statistical analysis between “Medium” and “Low” in Figure 1D was separately performed by t‐test due to an extensive range discrepancy in values among groups.

To study the specific roles of electrical conductivity in neural cell behaviors while decoupling other biologically influential parameters, we designed 2D carbon‐based materials.^[^ ^31^ ^]^ 3D conductive scaffolds that better mimic the native neural tissue environment while varying conductivity range were warranted as future work.^[^ ^32^, ^33^ ^]^ The electrical conductivity was precisely tuned independently of surface roughness, cell adhesive protein density, and substrate compositional element. Our chosen platform consisted of multi‐wall CNT and graphene oxide nanoribbon (GONR), which was subjected to an oxidation process (using KMnO_4_ and H_2_SO_4_) to precisely modulate the electrical conductivity (**Figure** **1**A).^[^ ^34^ ^]^ With increasing oxidation duration up to 48 h, the electrical conductivity progressively decreased from ≈ 800 to ≈ 0.02 S m^−1^ (Figure 1B). This decrease was linked to a reduction in free electrons, predominantly derived from the C─C bond in CNTs, which decreased from 62% to 46% due to the accumulation of oxygen functional groups, as revealed by XPS (Figure S1A, Supporting Information).

Upon the conductive substrates, we employed neuronal PC12 cells to evaluate the initial cell adhesion behavior. Notably, PC12 cells displayed significantly reduced cell adhesion on substrates with higher conductivity exceeding ≈ 4 S m^−1^, indicative of neurotoxicity (Figure S1B, Supporting Information). To determine the appropriate conductive range for our experiments, we compiled data from key recent reports that focused on neural (stem) cell lineage specification and neural regeneration, along with measurements of conductivity in native nerve tissues (Figure 1C). Approximately 63% (12/19) of these studies demonstrated enhanced neural differentiation in conditions mimicking native nerve tissue conductivity (0.03–0.6 S m^−1^).^[^ ^5^, ^35^, ^36^, ^37^, ^38^, ^39^, ^40^, ^41^, ^42^, ^43^, ^44^, ^45^, ^46^, ^47^, ^48^, ^49^ ^]^ However, other studies revealed a preference for neurogenesis on “supraphysiological” conductivity, despite the concerns of neurotoxicity.^[^ ^11^, ^43^, ^50^, ^51^, ^52^, ^53^, ^54^ ^]^ Many of these studies compared their results with non‐conductive tissue culture plates and failed to decouple key parameters governing neural cell fate specification, such as substrate composition, nanoscale roughness, and the quantity of cell adhesion proteins, while varying the conductivity. This led to an incomplete understanding of the role of electrical conductivity (Figure 1C).

Thus, we choose to encompass not only “native neural tissue‐like (≤ 0.6 S m^−1^)”, but also “supraphysiological (> 1 S m^−1^)” conductivity to gain a comprehensive understanding. To ensure that our initial cell adhesion assay did not involve neurotoxic conditions (i.e., oxidation duration less than 24 h), which might adversely affect cell fate determination, we selected “native nerve tissue” conductivity (“Medium (0.104 S m^−1^)” from 32 h oxidation and “Low (0.022 S m^−1^)” from 48 h oxidation) as well as “supraphysiological” conductivity (“High (3.22 S m^−1^)” from 24 h oxidation). These conditions were chosen while maintaining consistent compositions (carbon‐based), nanoscale roughness, and the amount of engrafted ECM protein, specifically laminin, on the substrates.

Raman spectrum analysis of the substrate surfaces revealed a slight increase in the intensity ratio of the D to G band, indicating reduced crystallinity within the graphitic layer due to increased oxidation.^[^ ^55^ ^]^ Additionally, high‐resolution X‐ray diffraction confirmed the presence of augmented oxidation functional groups from GONR within the “Medium” and “Low” substrates while decreasing peaks associated with MWCNT (Figure S2A and B, Supporting Information). The electrical conductivity of carbon substrates was assessed with a 4‐probe conductivity detection method with LED lighting, showing a gradual reduction in conductivity over oxidation (Figure 1D). We also conducted surface morphology and nano‐roughness assessments through field‐emission scanning electron microscopy and atomic force microscopy. These analyses confirmed the similarity in nano‐roughness across the various electroconductive carbon films (Figure 1E; Figure S2C, Supporting Information). Surprisingly, despite similar hydrophilicity (Figure 1F; Figure S2D, Supporting Information), a noticeable difference was observed in the absorbance of neuronal ECM protein, laminin. To address this discrepancy, we adjusted the pre‐coating material (poly‐D‐lysine) to ensure comparable laminin engrafting amounts among the groups (Figure 1G). Of note, the development of new conductive materials allowing direct neural cell adhesion could further enhance our understanding of the interplay between electrical conductivity and neural cell behavior. Stiffness, which can modulate stem cell fate, of bare substrates was similarly detected among the groups as 0.5–0.8 GPa, which is supraphysiologic compared to that of neural tissues (0.5–2 kPa) (Figure S3, Supporting Information).^[^ ^56^, ^57^ ^]^ For future studies, it would be beneficial to develop stiffness‐matched, hydrogel‐based conductive materials with tunable conductivity.^[^ ^58^ ^]^ In summary, we have successfully controlled the electroconductivity levels (from neural‐tissue‐like to supraphysiological) with good cell viability while decoupling other parameters, based on the oxidation degree using a carbon‐based platform. This allows us only to explore the potential effects of electrical conductivity (spanning from 0.02 to 3.2 S m^−1^), including the range found in native neural ECM, after decoupling other influential parameters that affect neural cell behaviors (Figure 1H).

### 2.2 Electroconductivity Resembling Neural Tissues Stimulates Neuronal Differentiation

To investigate the potential modulatory effects of electroconductive platforms on neural differentiation, we employed PC12 cells as an initial screening model due to their well‐established neurogenic response, while acknowledging the limitations of using a neuroblastoma‐derived cell line.^[^ ^59^, ^60^ ^]^ Contrary to prevailing assumptions about electrical conductivity, it was the neural tissue‐like conductivity (coded as “Medium” and “Low”), not supraphysiological conductivity (“High”), that demonstrated enhanced cellular adhesion and a greater number of neural branches at 24 h in the growth conditions (Figure S4, Supporting Information). This observation challenges the conventional belief that higher electrical conductivity is always a critical factor in designing neural scaffolds and interfaces. One recent report has highlighted that higher electrical conductivity is not always beneficial for the behaviors of cortical neurons, such as growth, maturation, and neuronal network activity, prompting us to investigate deeper into this phenomenon.^[^ ^12^, ^61^ ^]^

**Figure 2:**
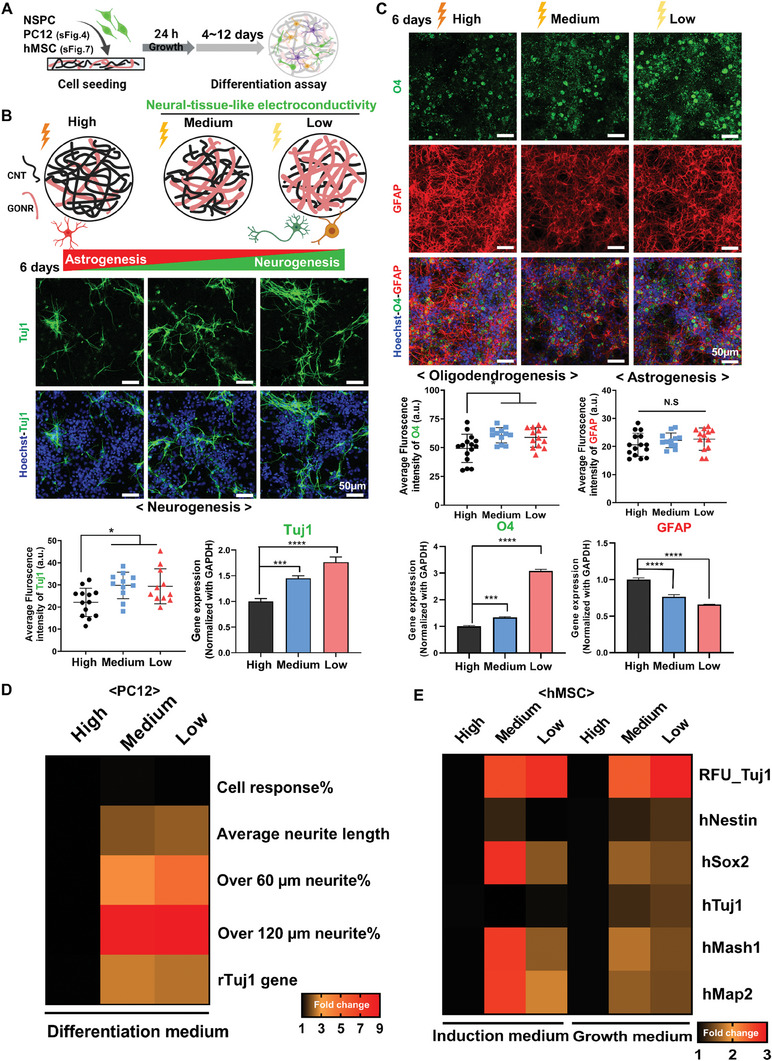
Neuronal differentiation is enhanced on neural tissue‐like electrical conductivity. A) A schematic figure illustrates the experimental procedure. B) The neuronal differentiation of rat NSPC was examined by immunostaining and gene expression of the neuronal maker Tuj1 after culture in the spontaneous differentiation conditions under growth factors withdrawal media for 6 days. C) The neuroglial differentiation of rat NSPCs by immunostaining and qPCR at differentiation day 6, including oligodendrocyte maker O4 and astrocyte maker GFAP. D) Heatmap from PC12 cell response and neurogenic gene expression under neurogenic differentiation under 100 ng mL^−1^ NGF after 4 days culture. All values are normalized to that of “High”. E) Heatmap from immortalized hMSC cell response and neurogenic gene expression under neuronal induction or growth medium. All values are normalized to that of “High”. RFU is a relative fluorescence unit from immunofluorescent staining. *P < 0.05, **P < 0.01, ***P < 0.001, ****P < 0.0001 (ANOVA and Turkey posthoc test after confirming normality and distribution symmetry by Shapiro‐Wilk test at a level of 0.05). N.S. indicated there was no significant difference between groups.

Next, we examined the effects of electrical conductivity on neurogenesis. For this, we employed spontaneous neural differentiation of NSPCs derived from rats without the addition of exogenous supplements, following the confirmation of NSPC characteristics on the tissue culture plate (**Figure** **2**A; Figure S5, Supporting Information). Before differentiation, NSPCs maintained stemness regardless of substrates (Figure S6, Supporting Information). After a 6‐day differentiation period, we evaluated the differentiation into the three representative neural lineages using markers Tuj1 (neuron), O4 (oligodendrocyte), and GFAP (astrocyte) (Figure 2B,C; Figure S7, Supporting Information). We observed accelerated differentiation toward neurons and oligodendrocytes (but not astrocytes) in the groups with lower conductivity when compared to the “High” conductivity group. Gene expression results corroborated these trends, exhibiting enhanced expression of Tuj1 and O4 genes with reduced astrogenic GFAPexpression in neural tissue‐like conductivity. Neurofilament staining at day 6 of differentiation confirmed enhanced neural differentiation on neural tissue‐like electrical conductivity (“Medium” and “Low”, Figure S8, Supporting Information). These findings align with another study demonstrating that higher electrical conductivity induced a higher propensity for astrogenesis in electroconductive hydrogels.^[^ ^13^ ^]^ In addition, our examination of conductivity‐dependent neuronal (not astrogenic) lineage specification at 12 days (Figure S9, Supporting Information) further confirmed the crucial role of conductivity in controlling neural stem cell specification over the course of several days.

To further validate our findings and explore the effects of electrical conductivity on human stem cells, we extended our examination to neuronal PC12 cells and immortalized human mesenchymal stem cells (MSCs),^[^ ^62^, ^63^, ^64^ ^]^ as the latter provide a relevant model for investigating human stem cell responses. Neurogenesis, including neurite growth, neurogenic elongated morphology, and the expression of neurogenic genes, was also enhanced in “Medium” and “Low” conductivity than the “High” counterpart under differentiation or neurogenic induction conditions. This reaffirms the pivotal role of electrical conductivity in guiding neurogenesis^[^ ^21^, ^65^ ^]^ (Figure 2D,E; Figures S10 and S11, Supporting Information). As aforementioned, the amount of ECM was found to impact neuronal differentiation in both pro‐neurogenic “Low” and less‐neurogenic “High” electroconductive substrates, emphasizing the importance of controlling the amount of ECM engrafted for investigation into the effects of electrical conductivity (Figures S12 and S13, Supporting Information). In summary, after decoupling other parameters from the electrical conductivity, we conclude that electrical conductivity governs neural cell lineage specification. Specifically, conditions resembling neural tissue‐like conductivity induce pro‐neurogenic cell responses rather than supraphysiological conductivity.

### 2.3 Differential Electrical Conductivity Leads to Distinct RNA Profiles Associated with Cell Fate

**Figure 3:**
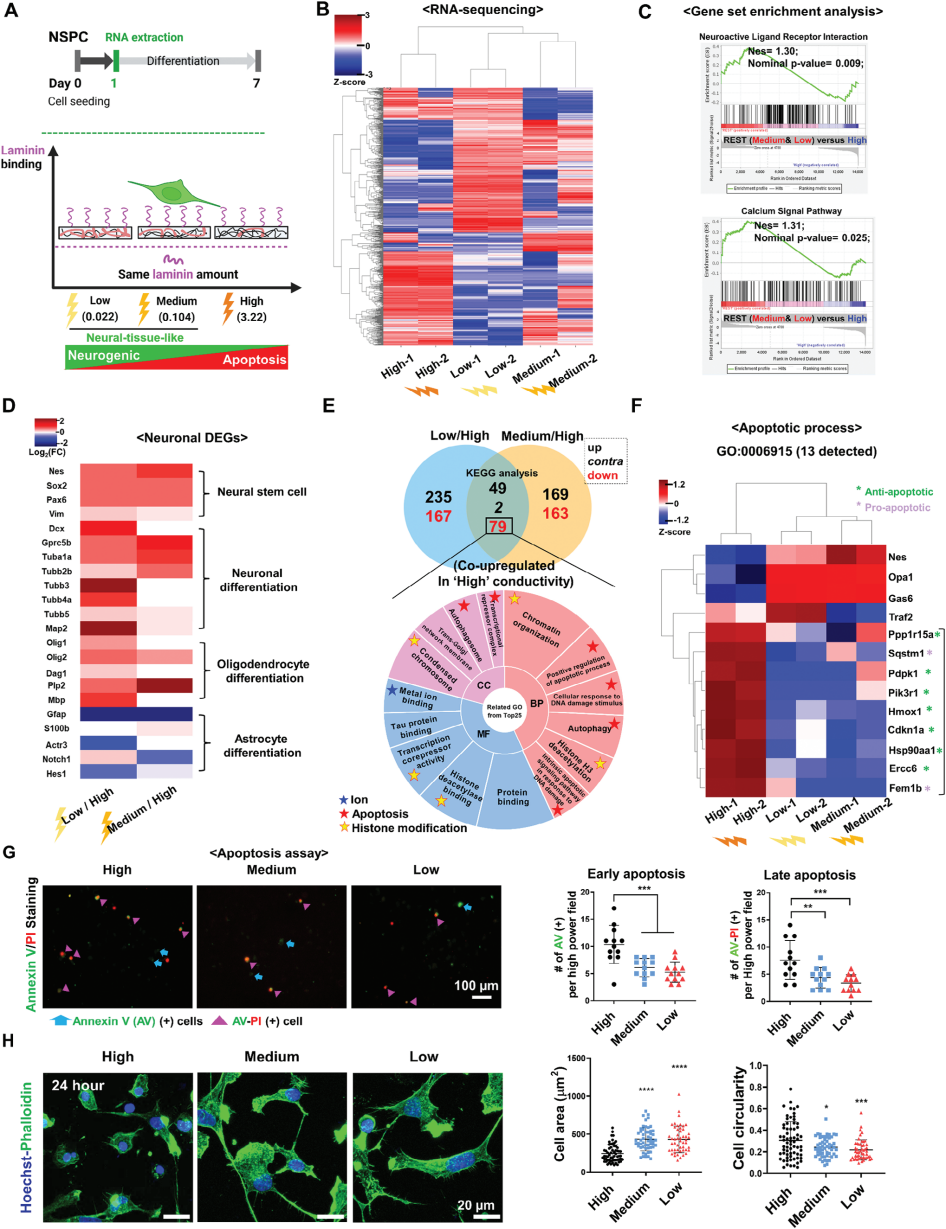
Transcriptomic analysis of NSPCs reveals distinct profiles in gene sets related to cell fate upon differential conductivity cues. A) The schematic figure shows the experimental timeline (top) and the strategy to compare the overall transcriptome of rat NSPCs under stemness‐maintaining media at 24 h based on the exogenous electroconductive property of carbon film substrates (bottom). B) Heatmap analysis of the DEGs (1.5 fold change) among different electrical conductivities. C) Gene Set Enrichment Analysis (GSEA) of the DEGs between the three different electrical conductivities. D) The representative neurogenesis (GO:0022008) related DEGs were summarized to show the neural transcription difference among different electrical conductivities. E). Gene Ontology (GO) analysis (bottom) for down‐regulated gene sets both in “Medium” and “Low” electroconductive carbon films as shown in the upper Venn diagram figure. F) The apoptotic process was further presented with a heatmap by analyzing the DEGs between the three electroconductive carbon film groups. G) Representative apoptotic NSPCs on different electrical conductivities at 24 h under stemness‐maintaining media. There was a significantly increased number of NSPCs undergoing the early (Annexin V) and late apoptosis (Annexin V‐PI) in the high electroconductive groups. H) Initial cell morphology of NSPCs cultured on the different electrical conductivities under stemness‐maintaining media at 24 h. The NSPCs cultured on the high conductivity displayed a shrunken cytoskeleton and less spreading behaviour. F‐actin (Green) and nucleus (blue). All the DEGs‐related analyses were derived from the DEGs under the setting of “Low” versus “High” conductivity, or “Medium” versus “High” conductivity, Fold change ≥ 1.5, Normalized value ≥ 2, n = 2. *P < 0.05, **P < 0.01, ***P < 0.001, ****P < 0.0001 (ANOVA and Tukey posthoc test after confirming normality and distribution symmetry by Shapiro‐Wilk test at a level of 0.05).

To gain insight into the behaviours of NSPCs on different electrical conductivity conditions, we conducted a comprehensive examination of overall transcriptional profiles after culturing for 24 h (**Figure** **3**A). An unsupervised heatmap was employed to visualize the profiles of differentially expressed genes (DEGs) across the spectrum of electrical conductivity. The profiles of “Low” and “Medium” revealed high similarity (Figure 3B). Gene set enrichment analysis identified “Calcium signal pathway (p = 0.025)” and “Neuroactive ligand‐receptor interaction (p = 0.009)” in the neural tissue‐like conductivity groups, which is consistent with the neurogenic differentiation capacity observed in “Low” and “Medium” (Figure 3C). Using DAVID Gene Ontology (GO) analysis, we focused on the co‐upregulated DEGs within the “Neurogenesis” category (GO 0022008) and observed several GO terms related to neural differentiation or calcium ion function among the top 10 biological processes, cellular component, and molecular functions in “Low” and “Medium” compared to “High”. These included terms like “Neuron differentiation”, “Oligodendrocyte differentiation”, “Dendrite morphogenesis”, “Neurogenesis”, “Neuroblast proliferation”, and “calcium ion binding” (Figure S14A, Supporting Information). A detailed heatmap of neuronal DEGs displayed elevated expression of gene sets related to “Neural stem cell”, “Neuronal differentiation”, and “Oligodendrocyte differentiation” in neural tissue‐like conductivity, along with decreased expression of gene sets related to “Astrocyte differentiation” (Figure 3D). Furthermore, genes from “Focal adhesion and ECM adhesion molecules” and pro‐astrogenic “Notch Signaling Pathway” displayed enhanced adhesion genes (e.g., FN1 – fibronectin and Anxa6 – annexin A6, a calcium‐dependent cell membrane protein) in “Low” and “Medium”, and notch signaling genes (e.g., dll1 – canonical notch ligand 1 and Fosl1 – FOS Like 1, a notch‐related transcription factor)) in “High”, repsectivly^[^ ^22^, ^23^ ^]^ (Figure S14B and C, Supporting Information). These findings collectively support that neural tissue‐like conductivity stimulates an increased propensity for neuronal differentiation.

To unravel the mechanistic explanation for why supraphysiological electrical conductivity induces fewer neurogenic effects, we conducted DAVID GO analysis on the co‐downregulated DEGs in “Low” and “Medium” compared to “High” (i.e., co‐upregulated in “High” compared to “Low” and “Medium”). The analysis revealed predominant associations with ion (likely calcium), histone modification, and apoptosis‐related GO terms, including “Metal ion binding”, “Chromatin organization”, “Histone H3 deacetylation”, “Histone deacetylase binding”, “Positive regulation of apoptotic process” and “Cellular response to DNA damage stimulus” (Figure 3E). These results prompted us to analyze in‐depth the gene sets within “Apoptotic process” GO category. This analysis unveiled higher expression of anti‐apoptotic genes (e.g., Ppp1r15a, Pdpk1, Pik3r1, Hmox1, Cdkn1a, Hsp90a1, and Ercc6) as well as pro‐apoptotic genes (e.g., Sqstm1 and Fem1b) in the “High” conductivity group (Figure 3F).^[^ ^24^, ^25^, ^66^, ^67^, ^68^ ^]^ These findings suggest that NSPCs on supra‐physiological “High” conductivity may be undergoing an apoptotic process. Consequently, the transcriptome analysis unveils the possibility of apoptotic behaviours of NSPCs as a potential anti‐neurogenic mechanism in response to supraphysiological conductivity (“High”).

To confirm the occurrence of apoptosis in NSPCs under the “High” conductivity condition, the Annexin V‐PI staining was conducted after 24 h of cell attachment. As shown in Figure 3G, there was a significant increase in the fraction of cells undergoing early apoptosis (≈10%, Annexin V‐positive) and late apoptosis (≈ 7.5%, Annexin V‐PI double‐positive) in the “High” conductivity group. Live and dead assay at 24 h confirmed more dead cells (lower cell viability) in “High” conductivity (Figure S15, Supporting Information). Cellular morphology was determined as another hallmark of apoptosis because the change in cell shape is indicative of different stages of apoptosis.^[^ ^69^, ^70^, ^71^ ^]^ Notably, NSPCs displayed reduced cell spreading area with rounded cell morphology (higher circularity) on “High” conductivity, which aligns with the observed increase in apoptotic cell fraction (Figure 3H).

### 2.4 Neural‐Tissue‐Like Conductivity Maintains Balanced Intracellular Calcium Dynamics, Unlike Supraphysiological Conductivity

**Figure 4:**
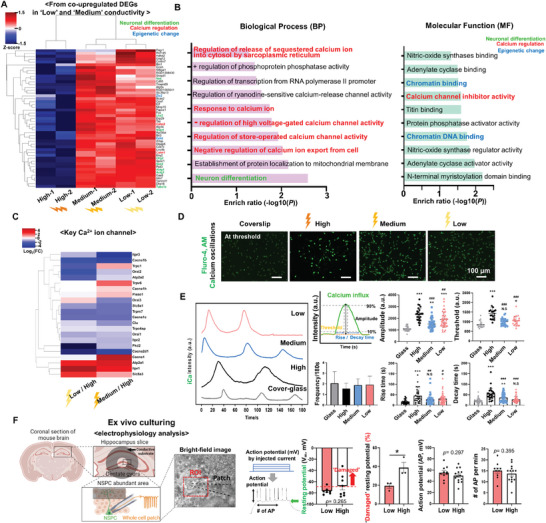
Neural tissue‐like electrical conductivity generates intracellular calcium dynamics different from the supraphysiological conductivity. A) Gene expression heatmap from co‐upregulated DEGs in “Low” and “Medium” compared to “High”. B) DAVID GO analysis from up‐regulated DEGs revealed neuron differentiation, intracellular calcium regulation, and chromatin DNA binding. C) A heatmap summarized the key calcium ion channel‐related DEGs. D) Representative fluorescence images of intracellular calcium (cell‐permeable Fluro‐4, AM, green) level of NSPCs under basal media at threshold state. E) Intracellular calcium oscillations analysis of NSPCs during 3 mins. Amplitude, threshold (basal level), frequency, and rise&decay time were quantitatively analyzed. F) Schematic diagram of ex vivo cerebellar slices incubating on different electrical conductivities and electrophysiology analysis by patch clamp. N.S, no significant, ^*^ *P* < 0.05, t‐test for two groups. For multiple groups, *P < 0.05, **P < 0.01, ***P < 0.001, ****P < 0.0001 (ANOVA and Tukey posthoc test after confirming normality and distribution symmetry by Shapiro‐Wilk test at a level of 0.05). For (E), * compared to glass, ^#^ compared to “High”.

To explore the underlying mechanism of how neural tissue‐like electrical conductivity promotes the NSPCs fate toward neurogenic lineage, we further examined the co‐upregulated DEGs in “Low” and “Medium” compared to “High” using DAVID GO analysis (Figure S16, Supporting Information). The analysis revealed genes associated with neuronal differentiation (Lhx2, Sox2, Nes, Olig2, Tuba1a, Actg1, and Map4), negative calcium regulation (calm1 and calm2), and chromatin DNA binding (Zic2 and Ezh2), along with related GO (**Figure** **4**A,B). Particularly, GO terms related to Ca^2+^ channels, such as “Calcium channel inhibitor activity”, “Regulation of the release of sequestered calcium ion into the cytosol by sarcoplasmic reticulum”, “Response to calcium ion”, “Negative regulation of high voltage‐gated calcium channel activity”, “Regulation of store‐operated calcium channel activity”, and “Negative regulation of calcium ion export from cell” were substantially enriched, prompting us to investigate Ca^2+^ channels. A specific analysis of Ca^2+^ channel‐related DEGs revealed the down‐regulation of key Ca^2+^ channels in both cell membranes (Orai1, Orai2, Slc8a1, Cacna1b, Cacna1c, Cacna2d1, Trpm7, and Pkd2) and endoplasmic reticulum (ER) (Itpr2 and Itrp3) in “Medium” and “Low” conductivity, suggesting a potential role of electrical conductivity in modulating Ca^2+^ channels (Figure 4C). Cumulative evidence has identified secondary messenger Ca^2+^ and Ca^2+^ channels as crucial regulators of fundamental processes in neuronal cell adhesion, differentiation, and apoptosis.^[^ ^72^, ^73^, ^74^ ^]^ Collectively, this suggests that intracellular Ca^2+^ ions may play a pivotal role in both neural differentiation and apoptotic behaviours of NSPCs in response to different electrical conductivity cues.

Next, we monitored intracellular Ca^2+^ dynamics using cell‐permeable Fluro‐4 AM for 3 min. At the basal threshold level, we observed a significant increase in intracellular calcium signaling in the “High” conductivity group, indicating prolonged activation of calcium ion influx (Figure 4D). This hyperactive calcium influx under the “High” conductivity condition was quantitatively characterized by higher amplitude and threshold, along with longer rise and decay time, without a change in frequency (Figure 4E; Figure S17A, Supporting Information). In silico intracellular calcium modeling on different conductivity also supported the above data (Figure S17B, Supporting Information). To date, cellular calcium overload and the subsequent disruption of intracellular calcium homeostasis are widely recognized as cytotoxic events, ultimately leading to apoptosis.^[^ ^75^, ^76^ ^]^ To explore whether live brain tissue also manifests this phenomenon, ex vivo patch clamp tests were conducted, involving the incubation of cerebellar slices, particularly the dentate gyrus, on donut‐shaped electroconductive substrates to maximize tissue‐substrate contact with patch clamp availability (Figure 4F). Notably, in contrast to the “Low” conductivity, a higher fraction of cells exhibited resting potential (> ‐70 mV) in “High” conductivity (≈ 40%) while other electrophysiological parameters remained unchanged. This shift in resting potential suggests that the electrical conductivity significantly impacts calcium ion homeostasis and subsequent biological processes at the tissue level.^[^ ^77^, ^78^ ^]^ For future studies, investigating specific calcium ion channels for governing conductivity‐mediated calcium influx is essential to gain a more comprehensive understanding.

### 2.5 Neural Physiological Conductivity Reprograms Chromatin by H3 Acetylation (H3ac) and Induces Neuronal Differentiation via Neurogenic Transcription Factor Activation

**Figure 5:**
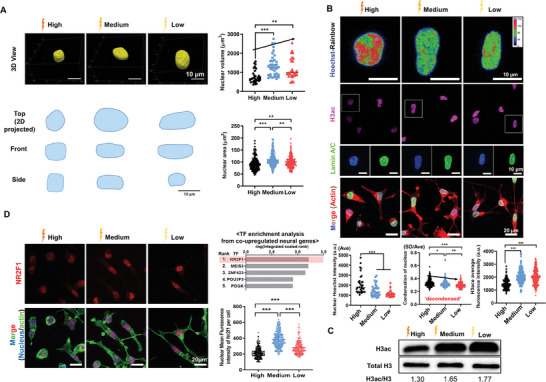
Neural tissue‐like conductivity reprogramms chromatin by H3 acetylation (H3ac) and induces neuronal differentiation of NSPCs via neurogenic transcription factor activation. A) 3D nucleus morphology and quantification on different electrical conductivities at 24 h under stemness maintaining media, revealing large 3D volume in neural tissue‐like conductivity as an indirect marker of nucleus decondensation. B) Represent fluorescence images of NSPCs' nucleus, H3ac, and lamin A/C, cultured on the different electrical conductivities under the stemness‐maintaining media at 24 h. The representative nucleus density was presented in the rainbow images to show the chromatin condensation condition. F‐actin (Alexa Fluor 546‐Phalloidin (red)), nucleus (Hoechst (blue)), Lamin A/C (red). Quantification of nucleus intensity, condensation factor, and H3ac intensity was shown. C) Representative Western blots of H3ac and total H3 from different conductivity under the stemness maintaining media at 24 h. D) The NR2F1 expression image and its semi‐quantification of NSPCs were presented as a representative electroconductivity‐dependent neurogenic transcription factor. After investigating transcription factor enrichment analysis using co‐upregulated genes (45 genes hit) in neural tissue like electroconductivity compared to the “High” counterpart from neurogenesis GO (GO:0022008, FC > 1.5, normalized data > 1, p‐value < 0.05), the top‐ranked NR2F1 was selected as a representative. *P < 0.05, **P < 0.01, ***P < 0.001 (ANOVA and Tukey posthoc test after confirming normality and distribution symmetry by Shapiro‐Wilk test at a level of 0.05).

Recent studies have revealed how extracellular cues can influence the nuclear shape, chromatin remodeling, and cytoskeleton components, ultimately altering lineage specification.^[^ ^79^, ^80^, ^81^, ^82^ ^]^ To investigate these changes, we visualized 3D nuclear morphology to analyze nuclear volume and projected morphology from top, front, and side views. Notably, under “High” conductivity, the nuclei of NSPCs appeared relatively smaller and rounder compared to the neural tissue‐like conductivity, suggesting chromatin compaction (**Figure** **5**A). As indicated in Figure 3E, GO terms related to histone 3 deacetylation (“Histone H3 deacetylation”, “Histone deacetylase binding”, and “Transcriptional repressor complex”) were identified from downregulated genes in “Low” and “Medium” conductivity, leading us to postulate an open chromatin status with increased H3ac in neural tissue‐like conductivity. Given that H3ac and H3K4me3 positively regulate transcription factors associated with neuronal differentiation,^[^ ^83^, ^84^ ^]^ we examined their expression levels and related epigenetic modifiers (Figure 5B; Figures S18–S20, Supporting Information). Compared to “High” conductivity, NSPCs cultured on the “Low” and “Medium” exhibited less nucleus intensity, greater decondensation, higher expression of euchromatic markers (H3ac and H3K4me3), and lower expression of heterochromatic markers (H3K9me3 and H3K27me3), possibly due to alterations in global and intricate epigenetic modifiers. Western blots supported higher H3ac expression in “Low” and “Medium” than “High”, reinforcing euchromatin status by global epigenetic change (Figure 5C).

Next, we sought to examine the interplay of these chromatin remodeling processes in modulating the transcription of neural genes and subsequently guiding the selective differentiation of NSPCs. We conducted an in‐silico search for potential conductivity‐dependent transcription factors (TFs) using the ChEA3 public database (Figure S21A, Supporting Information). When we assigned co‐upregulated genes in neural tissue‐like conductivity relative to “High” assigned to the neurogenesis GO (GO:0022008), we found enriched TFs such as CoupTF1 (Nr2f1), SOX11, SOX8, SOX2, and Pax6.^[^ ^85^, ^86^, ^87^, ^88^, ^89^ ^]^ Upon visualizing the top‐ranked neurogenic TF (Nr2f1) as a representative, it was evident that there was significant nucleus expression of Nr2f1 in the neural tissue‐like conductivity at 24 h after cell seeding, suggesting that less condensed chromatin (open) structures may induce subsequent neurogenic TF activation such as Nr2f1 (Figure 5D). When we examined other neurogenic TFs (Pax6 and Sox2), there were still distinguishable differences in nucleus expression among the groups, leaving open the possibility of multiple “conductivity‐dependent” TFs (Figure S21B–D, Supporting Information).

### 2.6 ATAC‐Seq Unveils Distinct Epigenetic Landscapes in NSPCs Cultured on Differential Electrical Conductivities

**Figure 6:**
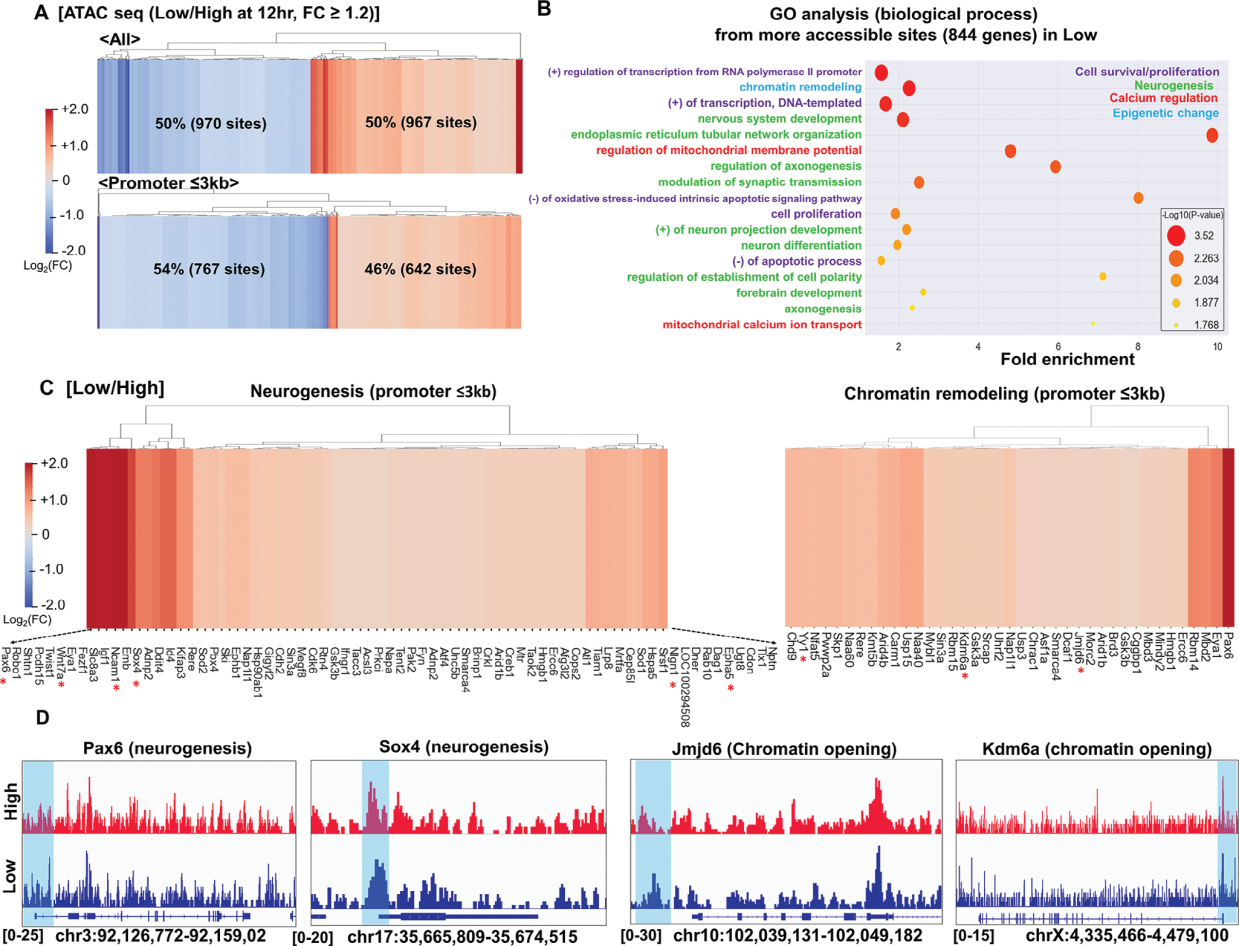
An assay for Transposase‐Accessible Chromatin with high‐throughput sequencing (ATAC‐seq) reveals distinct epigenetic modifications in NSPCs related to cell survival, neurogenesis, calcium regulation, and epigenetic change upon differential conductivity cues. ATAC‐seq was performed on NSPCs cultured on low and high conductive substrates for 12 h under stemness‐maintaining media. A) The analysis revealed the selective opening of promoter regions on low conductivity, as depicted in the heatmap for all and specific promoter regions. B) Gene ontology (GO) analysis connected differentially accessible genes to processes such as cell survival, neurogenesis, calcium regulation, and epigenetic change. C) The heatmap highlights the genes with increased DNA accessibility at promoter regions (≤3 kb) related to neurogenesis (Pax5, Wnt7a, Ncam1, Sox4, Nlgn1, Epha5) and chromatin remodeling to euchromatin status (Kdm6a, Jmjd6, Yy1) in low conductivity conditions. Key genes are highlighted as red asterisks. D) ATAC‐seq tracks for selected genes (Pax6, Sox4, Jmjd6, and Kdm6a) illustrate the differences in peak signal intensity (y‐axis) across DNA regions (x‐axis) between the two conductivity conditions (“High” versus “Low”).

To gain deeper insights into the conductivity‐induced chromatin remodeling, we employed an assay for transposase‐accessible chromatin sequencing (ATAC‐seq) to map chromatin accessibility in NSPCs cultured on low and high conductive substrates for 12 h under stemness‐maintaining conditions (Figure S22, Supporting Information). Intriguingly, NSPCs exhibited selective gains in overall (50% up versus 50% down) and promoter (46% up versus 54% down) accessibility on low conductivity substrates, with a fold change threshold of 1.2 (**Figure** **6**A). Gene ontology (GO) enrichment analysis revealed that the genomic regions upregulated in low conductivity conditions were associated with critical biological processes, including cell survival, neurogenesis, calcium regulation, and epigenetic modification (Figure 6B). The heatmap in Figure 6C highlights the genes with increased DNA accessibility at promoter regions (≤3 kb) related to neurogenesis (Pax5, Wnt7a, Ncam1, Sox4, Nlgn1, Epha5, etc.) and chromatin remodeling toward an euchromatin state (Kdm6a, Jmjd6, Yy1, etc.) in low conductivity conditions. The differential accessibility of promoter regions was further visualized using a genomics browser with ATAC‐seq tracks for representative neurogenesis (Pax6 and Sox4) and chromatin opening (Jmjd6 and Kdm5a) markers (Figure 6D). Notably, de novo motif analysis identified an enrichment of neurogenic transcription factors, such as Sox18, DMRT1, and Nur77, in low conductivity conditions (Figure S23, Supporting Information). Collectively, the ATAC‐seq analysis provides compelling evidence for conductivity‐mediated epigenetic changes, characterized by increased chromatin accessibility and the presence of neurogenesis‐favoring transcriptional regulators, in NSPCs cultured on more physiological, low conductivity substrates.

### 2.7 Intracellular Calcium Signaling and Chromatin Decondensation Explain the Enhanced Neuronal Differentiation upon Neural Physiological Conductivity

**Figure 7:**
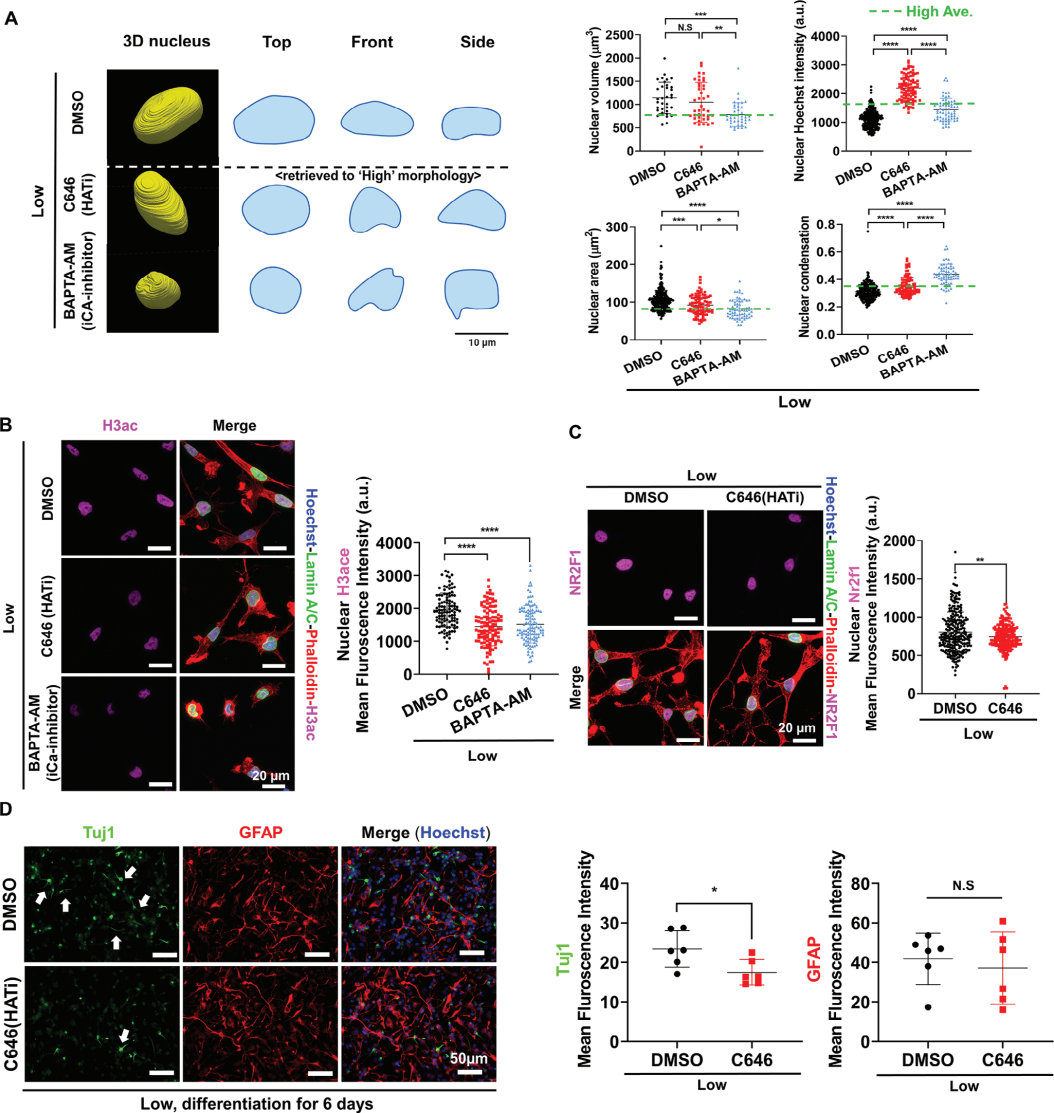
Chromatin reprogramming by H3 acetylation (H3ac) is key for neural tissue‐like conductivity‐dependent nuclear localization of NR2F1 and neurogenesis. A) The 3D nuclear morphology, including top, front, and side view, and its quantification with C646 (HATi, 10 µM) and BAPTA‐AM (intracellular calcium chelator, 10 µM) on “Low” conductivity. Dot green line indicates the average value of “High” conductivity. B) The nuclear H3ac activity was significantly decreased by C646 and BAPTA‐AM. Represent fluorescence images of the nucleus (blue), H3ac (violet), lamin A/C (green), and actin (red). NSPCs cultured on the different electrical conductivities were pre‐treated for 1 h at day 1 under the stemness‐maintaining media. C) The nuclear NR2F1 expression of NSPCs was reduced under C646 at day 1 under stemness maintaining media. D) The neuronal differentiation of NSPCs was partly diminished by the continuous treatment of HAT inhibitor C646 for 6 days. Neuronal maker Tuj1 (green), astrocyte marker GFAP (red), and nucleus (blue). White arrow indicating neuron cells with branching process. *P < 0.05, **P < 0.01, ***P < 0.001, ****P < 0.0001 (Student's t‐test or ANOVA and Tukey posthoc test after confirming normality and distribution symmetry by Shapiro‐Wilk test at a level of 0.05). N.S. indicated there was no significant difference between groups.

As demonstrated, we postulated that H3ac, in conjunction with balanced intracellular calcium ion dynamics, serves as the primary chromatin modifier that enhances the neuronal differentiation of NSPCs cultured on the neural tissue‐like conductivity; in contrast, the supraphysiological “High” conductivity triggers apoptotic process. To test our hypothesis, we administered the histone acetyltransferase (HAT) inhibitor C646 and the intracellular calcium chelator BAPTA‐AM specifically to NSPCs cultured on the “Low” conductivity, to ascertain the potential modulatory effects of H3ac and intracellular calcium dynamic on chromatin remodeling and cell fate specification. The nucleus area, DAPI intensity, and condensation were all retrieved to that of “High” conductivity by both inhibitors (BAPTA‐AM and C646), while the nuclear volume was reduced by only BABTA‐AM (**Figure** **7**A). Furthermore, the expression of H3ac, an euchromatin epigenetic marker, was also reduced by both inhibitors (Figure 7B). Taken together, we conclude that intracellular calcium‐mediated H3ac is a major regulator for tuning conductivity‐dependent nuclear and chromatin reprogramming. Subsequently, to determine the effect of chromatin reprogramming on neurogenesis, we monitored the nuclear localization of neurogenic TF (Nr2f1) and the neural differentiation behaviors of NSPCs upon the treatment of HAT inhibitor C646 for days 1 and 6, respectively. The C646 partly mitigated the initial nuclear activation of Nr2f1 at day 1 and consequently contributed to the reduction in neuronal differentiation of NSPCs at day 6 on the “Low” conductivity, without affecting astrogenesis (Figure 7C,D).

## 3 Concluding Remarks

**Figure 8:**
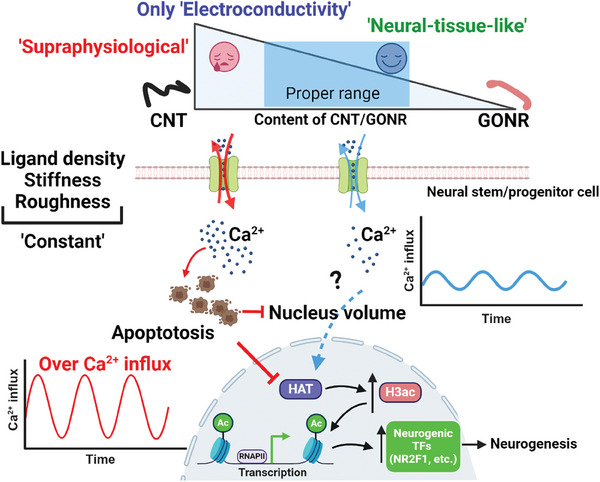
Schematic summary of supraphysiological or neural tissue‐like conductivity for modulating NPSC fate. H3ac, in conjunction with balanced intracellular calcium ions dynamics, serves as the primary chromatin modifier that enhances the neuronal differentiation of NSPCs cultured on the neural tissue‐like conductivity; in contrast, the supraphysiological “High” conductivity triggers apoptotic process while reducing nucleus volume and H3ac. How intracellular calcium dynamics alter the nucleus volume and HAT remains a further question (question symbol).

This study successfully designed a well‐controlled electrically conductive substrate platform utilizing functionalized carbon nanotubes and graphene oxide nanoribbons. The electroconductive substrates could maintain consistent surface composition, nano‐roughness, and cell‐binding protein density while varying only electrical conductivity. Within the limitation of the current carbon‐based material platform (2D microenvironment, need for protein coating, supraphysiologically high stiffness unlike native neural tissues), our observations revealed that supraphysiological conductivity (3.2 S m^−1^) hindered neuronal differentiation of neural stem/progenitor cells, due to the hyperactivated calcium signaling dynamics. In contrast, electrical conductivity resembling neural tissue‐like conditions (0.02–0.1 S m^−1^) prompted neuronal differentiation, with balanced intracellular calcium oscillation. Of note, this mild, neural tissue‐like conductivity induced changes in nuclear morphology and further alterations in chromatin structures toward a more decondensed status (increased histone 3 acetylation), which is also correlated with the nuclear localization of neurogenic transcription factors such as Nr2f1 (**Figure** **8**). Here, we prioritized investigating the downstream effects of calcium signaling on chromatin modifications and transcriptional regulation, rather than the upstream details of calcium ion channel regulation. Further experiments to identify the determinant calcium ion channels and validate the role of conductivity‐dependent neurogenic transcription factors, such as Nr2f1, are necessary for a deeper understanding of the mechanisms at play.

These findings highlight the intricate interplay between matrix electroconductive cues, nucleus morphology, and epigenetic modifications. Understanding the impact of electrical conductivity, under conditions decoupled from other substrate parameters, on nuclear morphology, chromatin accessibility, and cell fate specification, can provide valuable insights into the mechanisms in many biological contexts where the matrix conductivity‐triggered cellular responses (namely “electro‐transduction”) are essential. Further investigations are warranted to comprehensively elucidate the molecular mechanisms underlying these events, including the identification of candidate ion channels and the direct relationship between calcium signaling and chromatin modifications that drive neuronal specification. Moreover, this study offers guidance on how to design neural interfaces and scaffolds that can promote the regenerative process effectively.

## 4 Experimental Section

### Materials

*Preparations of CNT@graphene oxide nanoribbon bilayer membrane with various oxidation degrees*: The multiwall CNTs with different oxidation degrees were synthesized using a modified Hummer's method.^[^ ^30^, ^90^ ^]^ Briefly, 4 g of MWNTs (JenoTube 20A, JEIO, South Korea, diameter: 15–25 nm, length: 20–100 µm, and thickness: 7–12 layers) were mixed with 200 mL of sulfuric acid. Subsequently, 15.2 g of potassium permanganate (KMnO_4_) was carefully and slowly added to the mixture and stirred at 450 rpm in an ice bath. The mixture was then continued oxidation for 24 h (named High electroconductive group) in a water bath at 35 °C. Next, to stop the oxidation reaction and remove the remaining oxidizing agent, deionized (DI, 350 mL) water and hydrogen peroxide (80 mL) were sequentially added to the mixture in an ice bath. For making other electroconductivity, oxidation time varied from 1 to 48 h; 32 and 48 h are renamed “Medium” and “Low” electroconductive groups. Next, to remove the acidic solutions and impurities, the prepared CNT/GONR mixture with different oxidation degrees was filtrated through the cellulose filter paper using a vacuum and washed several times with DI water. Then, the filtered cake was ball‐milled at 800 rpm for 2 h in 60 mL of DI water and ball‐milled again at 350 rpm for another 3 h. The ball‐milled solution was sonicated for an additional 2 h using a horn sonicator to obtain the hydrogel form of CNT/GONR. At last, the bar‐coating method was used to fabricate carbon films. 0.5 mL of CNT@GONR hydrogel (40 mg mL^−1^) was coated on a polyethylene sulfone paper (PES, pore size: 0.2 m). The carbon films were dried in an oven at 60 °C for 3 h. The E.O. gas treatment sterilized the samples for the following cell works.

*Characterizations of carbon films*: A field emission‐scanning electron microscope (FE‐SEM; 7610f‐plus, JEOL, Japan) was used to examine the morphologies and structures of the prepared carbon films. The prepared carbon films' surface roughness and structure were investigated using atomic force microscopy (AFM; NX‐10, Park Systems, South Korea, n = 4). The phase of samples was confirmed by X‐ray diffraction (XRD, Rigaku, Ultima IV, Japan) with Cu Ka radiation at 40 mA and 40 kV, at a step size of 0.02° and a scanning speed of 2.0°/min. X‐ray photoelectron spectroscopy (XPS; K‐alpha, Thermo, U.K., USA), and Raman spectroscopy (LabRam Aramis, Horriba, Jovin Yvon, Japan) with a laser wavelength of 532 nm were utilized for surface characteristics. The electrical conductivity of the substrates was determined at room temperature by a four‐probe method using a Keithley 6514 electrometer.^[^ ^91^ ^]^ The hydrophilic ability of the films was analyzed by water contact angle test using a benchtop Phoenix contact angle measurement system (SEO‐PHX300, South Korea). Briefly, a single drop (10 µL) of DW loaded in a 1 mL syringe was dispensed onto the sample surface, and images of water droplets were captured after 10 s and further analyzed (n = 5). The specimen's stiffness (Elastic modulus) and contact stiffness were measured with a Nanoindenter (Nanoindenter XP MTS, Chicago, IL, USA). The Berkovich diamond tip roundness was 40 nm, the essential load was 1 mN, and the strain rate was maximally set at 200 nm s^−1^ with a penetration depth of 1 µm (n = 4). Loading (5 s), holding (2 s), and unloading (5 s) were performed.

### In Vitro Cell Studies

*Cell culture*: PC12 cells were ordered from ATCC company (CRL‐1721). PC12 cells are grown in RPMI‐1640 media (Welgene, LM 011‐03) with 5% FBS, 10% heat‐inactivated horse serum (Gibco, 26050088), and 1% (v/v) P/S solution. Every 2–3 days, the media were refreshed. The immortal human Mesenchymal stem cells were cultured in media used in Alpha MEM (Welgene, LM 008–53) supplemented with 10% (v/v) FBS, 1% (v/v) P/S, and 2 mM GlutaMAX supplement solution. This study was as follows: i) Normal culture medium composed of high‐glucose DMEM supplemented with 10% (v/v) FBS and 1% (v/v) P/S solution. Every 2–3 days, the media were refreshed. rNSPCs were prepared as previously reported.^[^ ^34^, ^92^, ^93^ ^]^ Animal experimental procedures for getting rNSPC were approved by the Animal Care and Use Committee, Dankook University, South Korea Approval No. DKU‐22‐071). Briefly, 14.5‐day embryos were isolated from pregnant Sprague‒Dawley rats (DBL Co., Ltd., South Korea). The telencephalons were isolated from embryos and placed in cold HBSS. The meninges of the telencephalon were removed, and the tissues were washed twice with cold HBSS and placed into 3 mL complete stemness maintenance media. The single‐cell suspension was dissociated in the stemness maintenance media using only one 1 mL pipette tip. After sitting for 2 minutes, the cell suspension was filtered with a 40 µm strainer, followed by centrifugation at 1500 rpm for 5 minutes. 5 × 10^5^ rNSPCs were seeded into 6 cm low‐attachment culture dishes and incubated in a 5% CO_2_ incubator at 37 °C. Half of the medium was changed every 2 days. The cells were passaged once per week when the sizes of the neuro‐spheroids were over 200 µm. In this study, cells were only used up to passage number 3. The stemness maintenance media contained knockout DMEM/F12,2 mMM L‐GlutaMAX, 2% Stempro neural supplement, 20 ng mL^−1^ EGF, 20 ng mL^−1^ bFGF, and 1% P/S.

### Cell Adhesion Assays

To assess the initial cell adhesion of PC12 and rNSPCs after similarly optimizing laminin adsorption amount, the high, medium, and low electroconductive carbon films were first incubated poly‐D‐lysine (15, 10, 15 µg mL^−1^) overnight at 4degree, respectively. After washing with PBS 3 times, 10 µg mL^−1^ cell adhesion protein laminin (Gibco, 23017015) was added and incubated for another night at 4 degrees. Then, 1.5 × 10^4^ cells were seeded only on the top of pretreated carbon films (a diameter of 8 mm) in 4‐well plates. After 1 h, more growth media were added to the cells. After 24 h, the cells were fixed using 4% PFA solution, permeabilized with 0.1% Triton X‐100 for 10 min and incubated for 30 min with Alexa Fluor 488 phalloidin (A12379, Invitrogen, Carlsbad, CA) and Hoechst 33 342 (1:1000; H1399, Invitrogen, Eugene, OR, USA) for 5 min to visualize actin filaments and nuclei, respectively. The stained images were captured using an Olympus DP72 camera (IX7151, Olympus Corporation, Tokyo, Japan). The adherent cell number, cell circularity, and cell spread area were further analyzed using ImageJ software (version 1.53, USA). To assess the initial cell adhesion of ihMSCs, 1.5 × 10^4^ cells were seeded only on the top of carbon films (a diameter of 8 mm) in 4‐well plates. After 1 h, more growth media were added to the cells. After 24 h, the cells were fixed using a 4% PFA solution, and the actin filaments and nuclei staining were similarly performed by that from PC12 and rNSPCs.

### Cell Differentiation Assays

To investigate the differentiation behavior of PC12, 2500 cells were seeded on the carbon films (a diameter of 8 mm). After 24 h, the media were refreshed with the RPMI media with 1% FBS, 1% horse serum, and 100 ng mL^−1^ NGF for another 4 days. At day 4, the PC12 cells were fixed with 4% PFA and permeabilized in PBS containing 0.1% (v/v) Triton X‐100 for 10 min. and incubated for 30 min with Alexa Fluor 488 phalloidin and Hoechst 33342 for 5 min to visualize actin filaments and nuclei, respectively.

For rNSPCs, 1.5 × 10^4^ cells were seeded on the carbon films (diameter 8 mm for ICC staining, diameter 10 mm for qPCR analysis) after coating with poly‐D‐lysine and laminin at the determined concentration depending on the conductivity. For tissue culture plate culture, 10 µg mL^−1^ of poly‐D‐lysine and laminin was used. After culturing in stemness maintaining media for 24 h, the media was refreshed with the same basal media but growth factors withdrawal. The rNSPCs were cultured in the differentiation media for another 6 or 12 days. Then, the samples were collected to perform the ICC staining or qPCR analysis. In ICC staining, after fixation and three washes in PBS, the cells were incubated in 1% (w/v) bovine serum albumin (BSA; SM‐BOV‐100, Gene all, Seoul, South Korea) dissolved in PBS for 1 h at RT to block nonspecific staining. Antibodies against βIII‐type tubulin (Tuj1; 801202, Bio Legend, CA, USA; dilution ratio: 1:100), anti‐O4 (MAB1326, R&D systems, MN, USA; dilution ratio: 1:250), GFAP (Dako Cytomation, Carpinteria, CA, Z0334; dilution ratio: 1:500), Tri‐Methyl‐Histone H3 (Lys9) (H3K9me3; 13969, Cell signaling, Danvers, MA, USA; dilution ratio: 1:100), Tri‐Methyl‐Histone H3 (Lys27) (H3K27me3; 9733, Cell signaling; dilution ratio: 1:100), Nestin (611 658, BD Bioscience, San Diego, CA, USA, dilution ratio: 1:100) and neurofilament (NF, 837904, Bio Legend, San Diego, CA, USA, dilution ratio: 1:100) were applied overnight at 4 °C, followed by three washes with PBS. Then, the cells were incubated in the dark with a 1:250 dilution of FITC‐conjugated donkey anti‐mouse IgG antibody (715‐095‐150, Jackson Immuno‐Research Laboratories, West Grove, PA, USA) or rhodamine (TRITC)‐conjugated antibody (715‐025‐150, Jackson) or Goat anti‐Mouse IgM (Heavy chain) Cross‐Adsorbed Secondary Antibody (Alexa Fluor 488, A‐21042; Invitrogen) for 1 h at RT. After three washes with PBS, nuclear staining was performed as mentioned above, and the different cell lineages were observed and captured using an inverted microscope (Olympus, IX71, Tokyo, Japan) and quantified using ImageJ.

### Gene Expressions by Quantitative Real‐Time PCR

Quantitative real‐time polymerase chain reaction (qRT‐PCR) was performed to evaluate the neural cells‐related gene expression. After cells were collected, mRNA was isolated using a Ribospin kit (GeneAll, 304‐150, Seoul, Korea) according to the manufacturer's instructions. cDNA was subsequently synthesized using AccuPower PCR premix (Bioneer, K‐2011, Daejeon, Korea), and the reverse transcription was performed using a thermal cycler (HID Veriti 96‐Well Thermal Cycler, 447 907, Applied Biosystems, Singapore). For qRT‐PCR, SensiMix SYBR Hi‐ROX kit (Bioline, QT605‐05) with additional MgCl_2_ (BIO‐37026, Bioline) was used, and qRT‐PCR performed by StepOne Plus (Applied Biosystems). The fold change of the gene expression was calculated by the comparative Ct method (2^−ΔΔCt^) and normalized to an endogenous housekeeping gene, β‐actin, or GAPDH. The qRT‐PCR primers and their sequences are listed in Table S1 (Supporting Information).

### Protein Expressions by Western Blots

Western blots were performed conventional method after culturing rNPSCs under growth media for 24 hours on films with different conductivity. Briefly, cellular lysates were directly prepared using Protein Extraction Solution (ELPISBIO, Daejeon, Korea, #EBA‐1149) supplemented with 1% Halt Protease and Phosphatase Inhibitor (ThermoFisher, Waltham, MA, #78 442), followed by a 30‐min incubation on ice. After the supernatant was collected after centrifugation, protein concentration was determined using Pierce BCA Protein Assay Kit (ThermoFisher, #23225). Subsequently, an equivalent amount of protein for each sample was mixed with Laemmli's 5× Sample Buffer (ELPISBIO, #EBA‐1052) and heated at 95 °C for 5 min. The mixture was then subjected to SDS‐PAGE electrophoresis. Afterwards, proteins were transferred onto Membranes, overnight incubation at 4 °C was conducted for the first antibody reactions. H3ac (Sigma, Burlington, MA, USA, 06–599, dilution ratio: 1:1000) and H3(Epigentek, Farmingdale, NY, USA, A68386, dilution ratio: 1:1000). Subsequently, membranes were probed with HRP‐conjugated anti‐mouse (Cell Signaling, #7074) or anti‐rabbit (Cell Signaling, #7076) secondary antibodies for 1 h. Detection was achieved using the Chemiluminescent Substrate Kit (ThermoFisher, #34580).

### Quantitative and ATAC Sequencing

For quantitative sequencing, 2.2 × 10^4^ rNSPCs were cultured upon carbon films (10 mm in diameter), pre‐sterilized by EO gas. After 24 h culture in stemness‐maintaining media, total RNA was isolated using RibospinTM (304‐150, Gene all, Seoul, South Korea) at 24 h according to the manufacturer's protocol. RNA purity was determined by assaying 1 µl of total RNA extract on a NanoDrop8000 spectrophotometer. Quantitative sequencing was then performed by Ebiogen Inc. (Seoul, South Korea). In brief, library preparation and sequencing were first performed. The library was constructed using the QuantSeq 3′ mRNA‐Seq Library Prep Kit (Lexogen, Inc., Austria) to control and test sample RNAs according to the manufacturer's instructions. An oligo‐dT primer containing an Illumina‐compatible sequence at its 5′ end was hybridized to the RNA (each 500 ng total RNA), and reverse transcription was performed. After degradation of the RNA template, second strand synthesis was initiated by a random primer containing an Illumina‐compatible linker sequence at its 5′ end. To purify the double‐stranded library, magnetic beads were used to remove all reaction components. The library was amplified to add the complete adapter sequences required for cluster generation. The finished library was purified from PCR components. High‐throughput sequencing was performed as single‐end 75 sequencing using NextSeq 500 (Illumina, Inc., USA).

Subsequently, QuantSeq 3′ mRNA‐Seq reads were aligned using Bowtie2.^[^ ^94^ ^]^ To align to the genome and transcriptome, Bowtie2 indices were either generated from genome assembly sequences or representative transcript sequences. The alignment file was used for assembling transcripts, estimating their abundances, and detecting the differential expression of genes. DEGs were determined based on counts from unique and multiple alignments using coverage in Bedtools.^[^ ^95^ ^]^ The read count (RC) data were processed based on the TMM+CPM normalization method using EdgeR within R (R development Core Team, 2020) using Bioconductor (Gentleman et al., 2004). The raw data were manually analyzed using ExDEGA software (v1.6.7, E‐biogen). Gene classification was further analyzed in the public database (DAVID (http://david.abcc.ncifcrf.gov/) and Medline databases (http://www.ncbi.nlm.nih.gov/)). The QuantSeq data are available at the NCBI Gene Expression Omnibus with accession number GSE241362 (https://www.ncbi.nlm.nih.gov/geo/info/ linking.html). The ChEA3 public database was used to analyse TFs (https://maayanlab.cloud/chea3/).^[^ ^96^ ^]^

ATAC‐sequencing was performed and analyzed by conventional methods according to the protocols mentioned elsewhere^[^ ^97^, ^98^ ^]^ [ref]. Briefly, 2.2 × 10^4^ rNSPCs were cultured upon each carbon film (10 mm in diameter), pre‐sterilized by EO gas. After 12 h's culture in stemness‐maintaining media, cells are gathered by TrypLE (Thermofisher, Philadelphia, PA, USA) for the construction of Library using ATAC‐Seq Library Prep Kit for Illumina (Active Motif, United States) according to the manufacturer's instructions. A total of 100,000 cells were washed twice with 100 𝜇l of cold PBS and resuspended in 100 𝜇l of STEM‐CELLBANKER solution (Amsbio). Next, 100 𝜇l of lysis buffer for 10 min in ice to prepare the nuclei. Immediately after lysis, the suspension of nuclei was spun at 500 g at 4 °C for 10 min to remove the supernatant. Nuclei were then incubated with 50 ul of the Tagmentation Master Mix at 37 °C for 30 min in a thermomixer set at 800 rpm. DNA was purified and PCR was performed to amplify the library. Libraries were sequenced on the NovaSeq 6000 with 100 bp paired‐end reads (Illumina, United States).

### Ex‐Vivo Brain Slice Analysis

Hippocampal slices were prepared from 5–8 weeks‐old C57BL/6 mice. Under isoflurane anaesthesia, the brain was quickly removed from the skull and placed in ice‐cold artificial cerebrospinal fluid (ACSF) aerated with 95% O_2_ and 5% CO_2_. The ACSF composition was as follows (in mM): 124 NaCl, 1.3 MgSO_4_, 3 KCl, 1.25 NaH_2_PO_4_, 26 NaHCO_3_, 2.4 CaCl_2_, and 10 glucose. After chilling in the ice‐cold ACSF for ≈3–5 min, the brain was sliced into hippocampal sections (300 µm thickness) using a vibroslicer (5100mz, Campden Instruments, UK). The slices that included the dentate gyrus were selected and kept in aerated (95% O_2_/5% CO_2_) ACSF at 35 °C for 60 minutes before being transferred to an electrophysiological recording chamber. Animal experiments for ex‐vivo study were performed in accordance with Dankook University Animal Experimentation Guidelines (approval number DKU‐19–016, Cheonan, Korea).

Recording the electrophysiological properties of the slices was carried out using whole‐cell patch‐clamp techniques with a differential interference contrast (DIC) microscope (Eclipse FN1, Nikon, Japan), Multiclamp 700B, and Digidata 1550B (Molecular Device, USA) as described previously.^[^ ^99^ ^]^ The slices were perfused with warm ACSF (34–35 °C) at a rate of ≈ 2 mL min^−1^ using a gravity‐fed bath perfusion system. Micropipettes (6–9 MΩ), pulled from borosilicate capillaries using a micropipette puller (PC‐10, Narishige, Japan) and filled with intracellular solutions, contained (in mM): 140 K‐gluconate, 10 HEPES, 2 MgCl_2_, 1 CaCl_2_, 11 EGTA, and 2 K2‐ATP. The pH was adjusted to 7.2–7.3 with KOH. A 10 mm diameter donut‐shaped substrate with a 3 mm inner defect and different electroconductivity (“High” or “Low”) was placed on top of the slice for electrophysiological recording of a single cell, considered as NSPC. Pipette capacitance was neutralized, and access resistance was continuously monitored during recording. In whole‐cell current‐clamp recordings, depolarizing step currents ranging from −50 to 200 pA were used to trigger action potentials at 10 pA increments and a duration of 300 ms. In whole‐cell voltage‐clamp recordings, whole‐cell currents were triggered with voltage steps ranging from −50 to 100 mV at 10 mV increments. Data analysis was performed using pClamp 10.0 (Molecular Device, USA).

### In Silico Intracellular Calcium Level Modeling

A simple mathematical model was designed to explain the influence of electroconductivity on the calcium dynamics. The model consists of ordinary differential equations with two variables: the calcium ion concentration of the cytoplasm and ER^85^. The detailed equations and parameters are shown in **Table** **1**. As membrane charging time strongly depends on extracellular electroconductivity and typically decreases when the extracellular electroconductivity increases, the calcium ion channel was also assumed to be impacted by the following changes. The result shows that as electroconductivity becomes higher, the calcium intensity of the cytoplasm also increases.

**Table 1:** Parameters and equation for intracellular calcium level modeling.

Parameter	Value (Control (ES))	Description
β	2.1 / 2.0 / 1.9 s^−1^	Rate of calcium efflux from cytosol to extracellular
α	1.3 s^−1^	Maximum rate of calcium flux into cytosol
γ	2.1 / 2.0 / 1.9 µM s^−1^	The inward flow of calcium from extracellular to cytosol through the plasma membrane
*n*	4	Hill coefficient
δ	1.3 µM	Cytosolic IP_3_ concentration that produces half occupation of IP_3_R
*k*	0.01 s^−1^	Leak flux rate of calcium from ER to cytosol due to concentration gradient
*k*_1_	2 s^−1^	Maximum flux rate of calcium out of the SERCA pump
Symbol	Equation	
[Ca^2+^]*_i_*	$k{\left[Ca^{2+}\right]}_{\mathrm{ER}}-k_{1}{\left[Ca^{2+}\right]}_{i}+\alpha \left(\frac{{\left[Ca^{2+}\right]}_{i}^{n}}{\left(\delta^{n}\right)+\left(\;{\left[Ca^{2+}\right]}_{i}^{n}\right)}\right)*{\left[Ca^{2+}\right]}_{\mathrm{ER}}+\gamma -\beta *{\left[Ca^{2+}\right]}_{i}$	
[Ca^2+^]_ER_	$-(k{\left[Ca^{2+}\right]}_{\mathrm{ER}}-k_{1}{\left[Ca^{2+}\right]}_{i}+\alpha \left(\frac{{\left[Ca^{2+}\right]}_{i}^{n}}{\left(\delta^{n}\right)+\left(\;{\left[Ca^{2+}\right]}_{i}^{n}\right)}\right)*{\left[Ca^{2+}\right]}_{\mathrm{ER}})$	

### Statistics

Data are presented as mean ± standard deviation, and statistical significance was assessed using Student's t‐test (between two groups) or one‐way analysis of variance (ANOVA) with an unpaired t‐test or Turkey's post hoc test (among over three groups) after confirming normality and distribution symmetry by Shapiro‐Wilk test at a level of 0.05. Statistical significance was considered at ^*^ *P* < 0.05, ^**^ *P* < 0.01, ^***^ *P* < 0.001 or ^****^ *P* < 0.0001. Independent triplicate experiments were at least performed to verify reproducibility. Careful interpretation of the results with relatively small numbers (n < 10) was needed.

## Conflict of Interest

The authors declare no conflict of interest.

## Author Contributions

Y.‐M.L., Y.J., and Y.‐X.M. contributed equally to this work as co‐first authors. Y.‐M.L., Y.‐X.M., Y.J., J.‐H.L., and H.‐W.K. conceptualized the study and designed the experiments; J.‐H.L. and H.‐W.K. supervised the study; Y.‐M.L., Y.‐X.M., Y.C., Y.‐J.K., A.G.K. J.‐H.P, J.‐Y.Y., H.L., R.K.S., J.C.K., H.‐H.L., Y.J., J.‐H.L. performed characterized the samples and analyzed the data. Y.‐M.L., Y.‐X.M., E.‐S.K., B.‐E.Y., and J.‐H.L. performed the in vitro cell and ex vivo studies. Y.‐M.L., Y.‐X.M., Y.J., and J.‐H.L. drafted the manuscript; H.‐W.K. and J.‐H.L. wrote, edited, and approved the manuscript.

## Supporting information

Supporting Information

## Data Availability

The data that support the findings of this study are available from the corresponding author upon reasonable request.
