# Supplementary material for: Neural Tissue‐Like, not Supraphysiological, Electrical Conductivity Stimulates Neuronal Lineage Specification through Calcium Signaling and Epigenetic Modification
Source: Adv Sci (Weinh). 2024 Jul 10;11(35):2400586. doi: 10.1002/advs.202400586 (PMC11425260; doi:10.1002/advs.202400586)
Supplement: Supplementary file 1 — Supporting Information [file ADVS-11-2400586-s001.docx]

**Neural-tissue-like, not supraphysiological, electrical conductivity stimulates neuronal lineage specification through calcium signaling and epigenetic modification**

Yu-Meng Li^1,2,#^, Yunseong Ji^1,3,#^, Yu-Xuan Meng^1,2,#^, Yu-Jin Kim^4^, Hwalim Lee^1,4^, Amal George Kurian^1,2^, Jeong-Hui Park^1,5^, Ji-Young Yoon^1,2,^, Jonathan C. Knowles^1,2,5,6^, Yunkyu Choi^7^, Yoon-Sik Kim^1,8,11^, Bo-Eun Yoon^1,8,11^, Rajendra K. Singh^1,2^, Hae-Hyoung Lee^1,2,4^, Hae-Won Kim ^1,2,4,5,8,9,10*^, Jung-Hwan Lee^1,2,4,5,8,9*^

^1^Institute of Tissue Regeneration Engineering (ITREN), Dankook University, Cheonan, Chungcheognam-do, 31116, Republic of Korea

^2^Department of Nanobiomedical Science and BK21 Four NBM Global Research Center for Regenerative Medicine, Dankook University, Cheonan, Chungcheognam-do, 31116, Republic of Korea

^3^Fuel Cell Laboratory, Korea Institute of Energy Research (KIER), Daejeon 34129, Republic of Korea.

^4^Department of Biomaterials Science, College of Dentistry, Dankook University, Cheonan, Chungcheognam-do, 31116, Republic of Korea

^5^UCL Eastman-Korea Dental Medicine Innovation Centre, Dankook University, Cheonan, Chungcheognam-do, 31116, Republic of Korea

^6^Division of Biomaterials and Tissue Engineering, UCL Eastman Dental Institute, Royal Free Hospital, Rowland Hill Street, London, NW3 2PF, UK

^7^Department of Chemical and Biomolecular Engineering Yonsei University, Seoul, 03722, Republic of Korea

^8^Mechanobiology Dental Medicine Research Center, Dankook University, Cheonan, Chungcheognam-do, 31116, Republic of Korea

^9^Cell & Matter Institute, Dankook University, Cheonan 31116, Republic of Korea

^10^Department of Regenerative Dental Medicine, College of Dentistry, Dankook University, Cheonan, Chungcheognam-do, 31116, Republic of Korea

^11^Department of Molecular Biology, Dankook University, Cheonan 31116, Republic of Korea

----------------------

#Equally contributed as co-first authors.

*Corresponding authors:

- Jung-Hwan Lee (ducious@gmail.com and ducious@dankook.ac.kr)

- Hae-Won Kim ([kimhw@dku.edu](mailto:kimhw@dku.edu))

- Address: Institute of Tissue Regeneration Engineering (ITREN), Dankook University, Republic of Korea


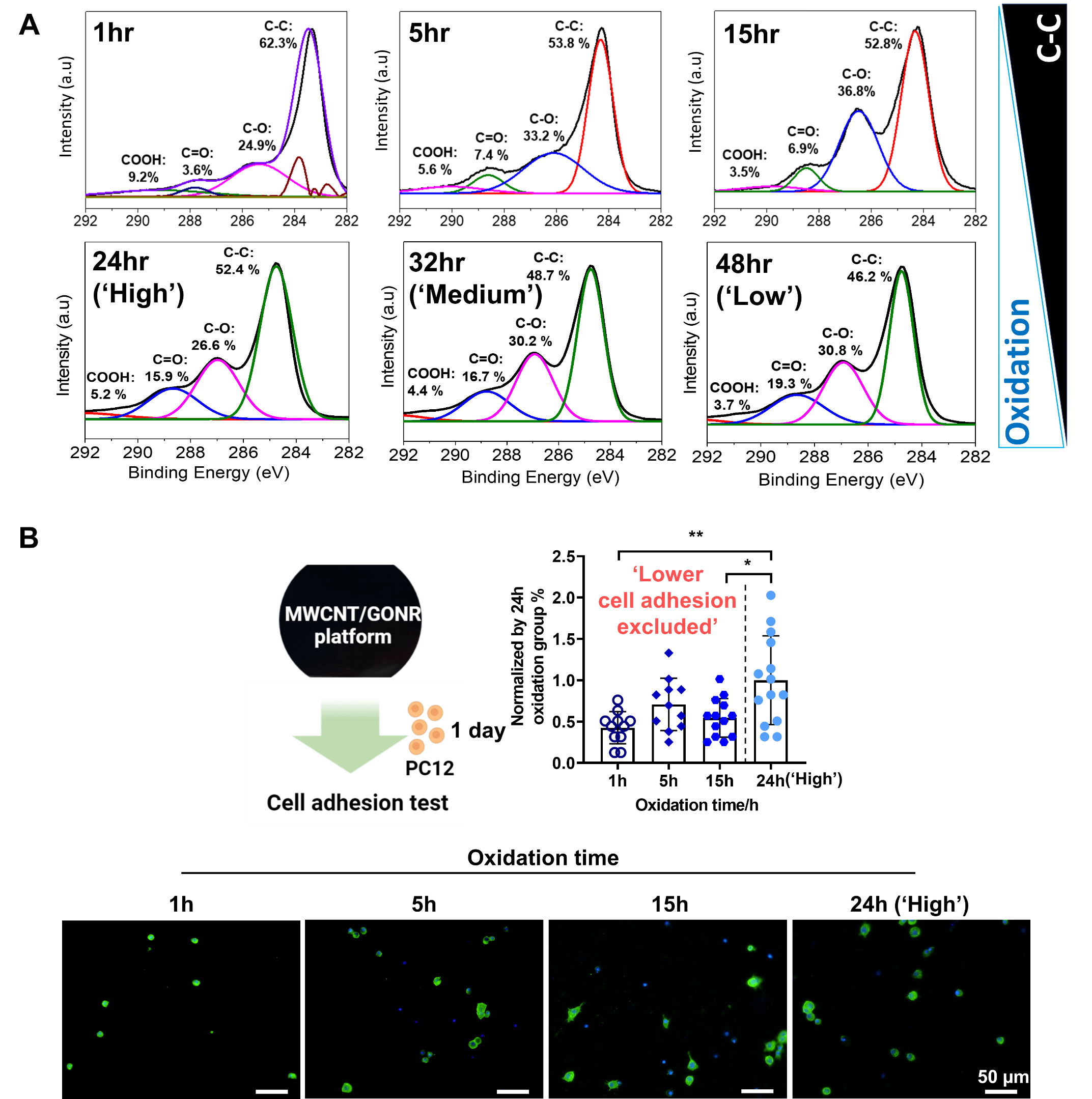


**Figure.S1. Electroconductive property and initial 24-hour PC-12 cell adhesion condition of different conductive carbon films.** (A-B) The initial cell growth condition of PC-12 cells cultured on carbon films, including 1-24 h oxidation time groups. (A) With the oxidation time increasing, the c-c binding decreased along with reduced electroconductivity, investigated by XPS. (B) Adherent cell numbers were compromised in 1h~15h oxidation substrate. The cytoskeleton maker F-actin was labelled with Alexa Fluor 488-Phalloidin (Green). The nucleus was marked with Hoechst in blue colour. ^*^*P* < 0.05 and ^***^*P* < 0.001 (ANOVA and tukey posthoc test after confirming normality and distribution symmetry by Shapiro-Wilk test at a level of 0.05). XPS data from 1, 5, and 10 hours were obtained from doi/10.1021/acsami.1c24733 and re-analyzation was performed.

**
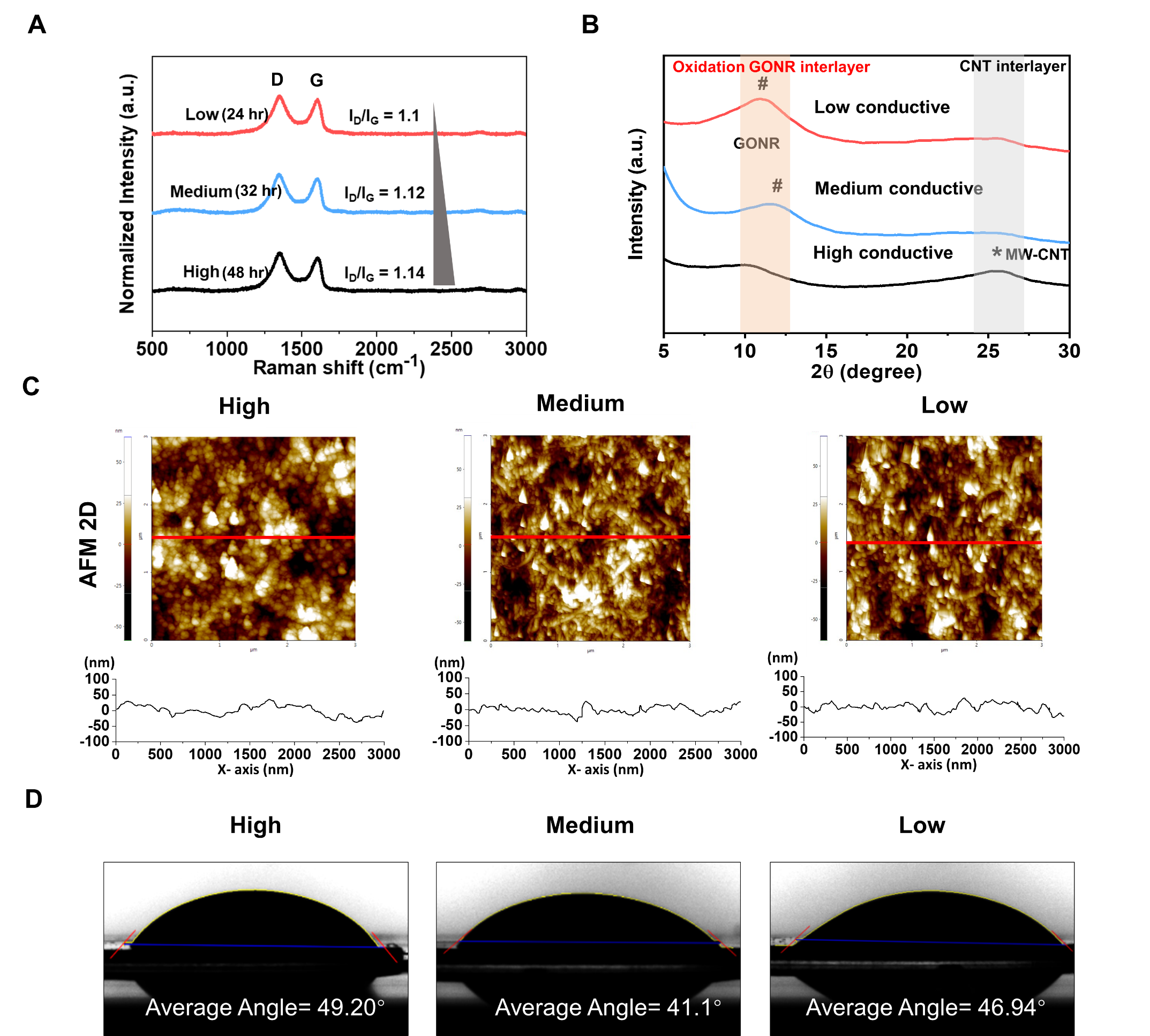
**

**Figure.S2. Physicochemical characterization of different conductive carbon films.** (**A**) Raman spectra revealed a slight increase in the intensity ratio of the D to G band, indicating reduced crystallinity within the graphitic layer due to increased oxidation. (**B**) High-resolution X-ray diffraction confirmed an augmentation in the oxidation functional groups from GONR within the ‘Medium’ and ‘Low’ while decreasing MWCNT peaks. (**C**) Similar surface nano-roughness in the nm scale was observed along the specific profile from the red line in the AFM 2D pictures. (**D**) A water contact angle test was performed to check the hydrophilicity of carbon films.

**
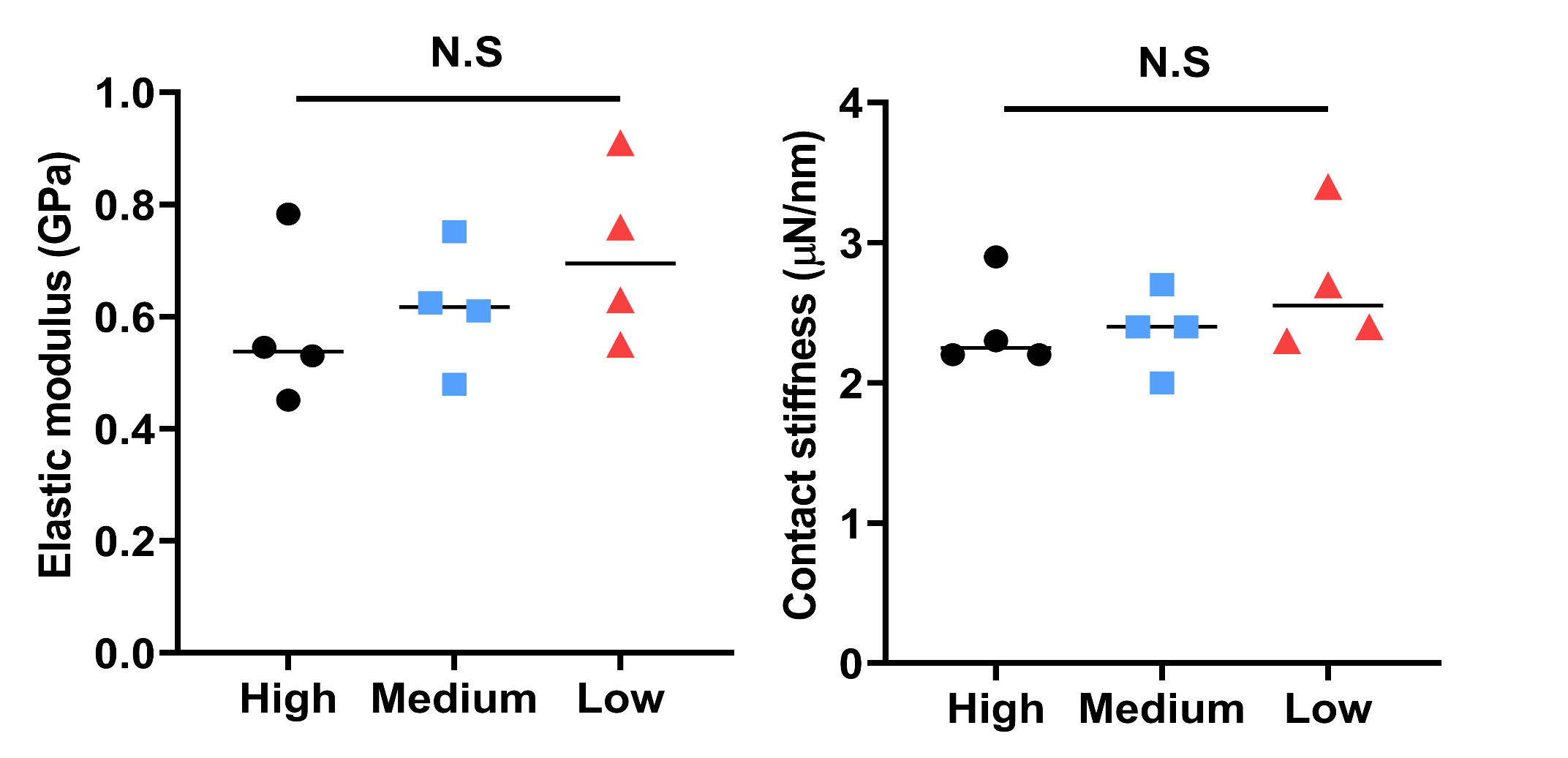
**

**Figure.S3. Stiffness of substrate measured by nanoindentor.** Elastic modulus and contact stiffness were similarly detected among groups (P>0.05, ANOVA and tukey posthoc test after confirming normality and distribution symmetry by Shapiro-Wilk test at a level of 0.05). N.S. indicated there was no significant difference between groups.

**Table. S1. Summary table related to the recently published electroconductive scaffolds within the field of neural regeneration.**

| **Electroconductive biomaterials in the neural field** | | **Conductivity (S/m)** |
| --- | --- | --- |
| Samples in this study | High conductive | 3.22 |
|  | Medium conductive | 0.104 |
|  | [Low conductive](mailto:PUCL@CNT1%20nanofibers) | 0.022 |
| CNTs-based conductive scaffolds | CNT@PUCL scaffolds ^[1]^ | 8 |
|  | CNTs-decorated PLGA scaffold ^[2]^ | ∼ 227 |
|  | CNT-interfaced PGFs scaffold ^[3]^ | 1 x10^-3^ - 1×10^-4^ |
|  | [rolled PVDF@CNT/BaTiO3 scaffold](mailto:CNT@BaTiO3@PVDF%20substrates) ^[4]^ | 10^–9^ - 10^-2^ |
|  | [CNTs-PCL/Gelatin nanofiber platform](mailto:MWCNTs@Chitosan%20scaffold) ^[5]^ | ~ 0.15 S/m |
| Graphene-based conductive scaffolds | [GO@oligo (poly (ethylene glycol) fumarate) (OPF) hydrogel](mailto:GO@oligo(poly(ethylene%20glycol)%20fumarate)%20(OPF)%20hydrogel) ^[6]^ | 0.09 - 0.4235 |
|  | reduced GO/PCL film substrate ^[7]^ | 5.5 - 7.1×10^-4^ |
|  | rGO/PCL scaffolds ^[8]^ | 0.84 - 1.47 |
|  | GO/PCL nano-scaffolds ^[9]^ | 0.0455 |
|  | graphene-based conductive fibrous scaffold ^[10]^ | 3.12 |
|  | rGO ApF/PLCL nanofibrous scaffolds ^[11]^ | 0.0405 |
| Other conductive scaffolds | Polyaniline (PANI) film substrate ^[12]^ | 10^-7^ - 1000 |
|  | Polypyrrole/silk fibroin scaffold ^[13]^ | 1.82 ± 0.21 × 10^-3^ - 0.113 ± 0.19 |
|  | polypyrrole (PPy) /micro-grooved PLGA scaffolds ^[14]^ | 963-3856 |
|  | polycaprolactone and chitosan@Au-NPs scaffolds ^[15]^ | 12 |
|  | collagen/ppy-b-PCL hydrogel ^[16]^ | 0.5 |
|  | Hyaluronic acid/gelatin/gold nanorod hydrogels ^[17]^ | 0.00115 |
|  | Polyaniline graphene (PAG)/chitosan/PCL scaffold ^[18]^ | 0.0108 |
|  | Ppy@collagen/hyaluronan hydrogel ^[19]^ | 0.189 |
| Native nerve tissues | Dura, grey matter, white matter, spinal cord ^[20–23]^ | 0.03~0.6 |

**
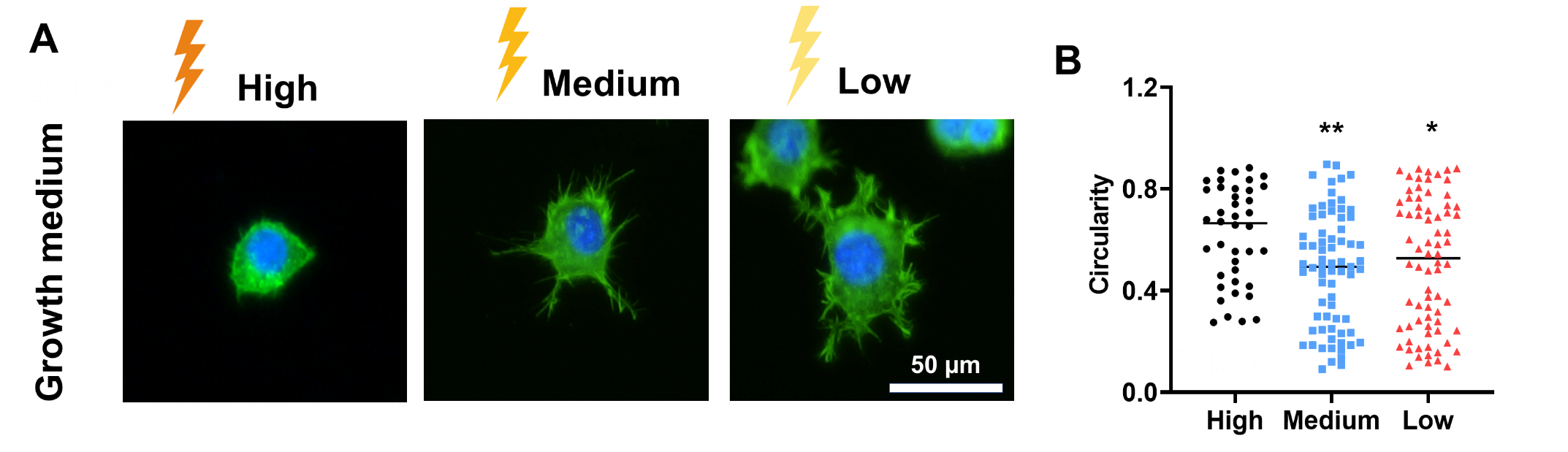
**

**Figure.S4.** **PC-12 cells exhibited better adhesion on the less conductive carbon film group.** (A) PC-12 cells exhibited different morphology depending on the electroconductivity under the growth media at 24 hours. Representative morphology was visualized (nucleus (blue dapi) and actin (green phalloidin)). (B) Initial adhesion and morphological responses of PC-12 cells cultured on the different conductive carbon films at 24 hours under growth media. ^*^*P* < 0.05 and ^**^*P* < 0.01 compared to High (ANOVA and tukey posthoc test after confirming normality and distribution symmetry by Shapiro-Wilk test at a level of 0.05).


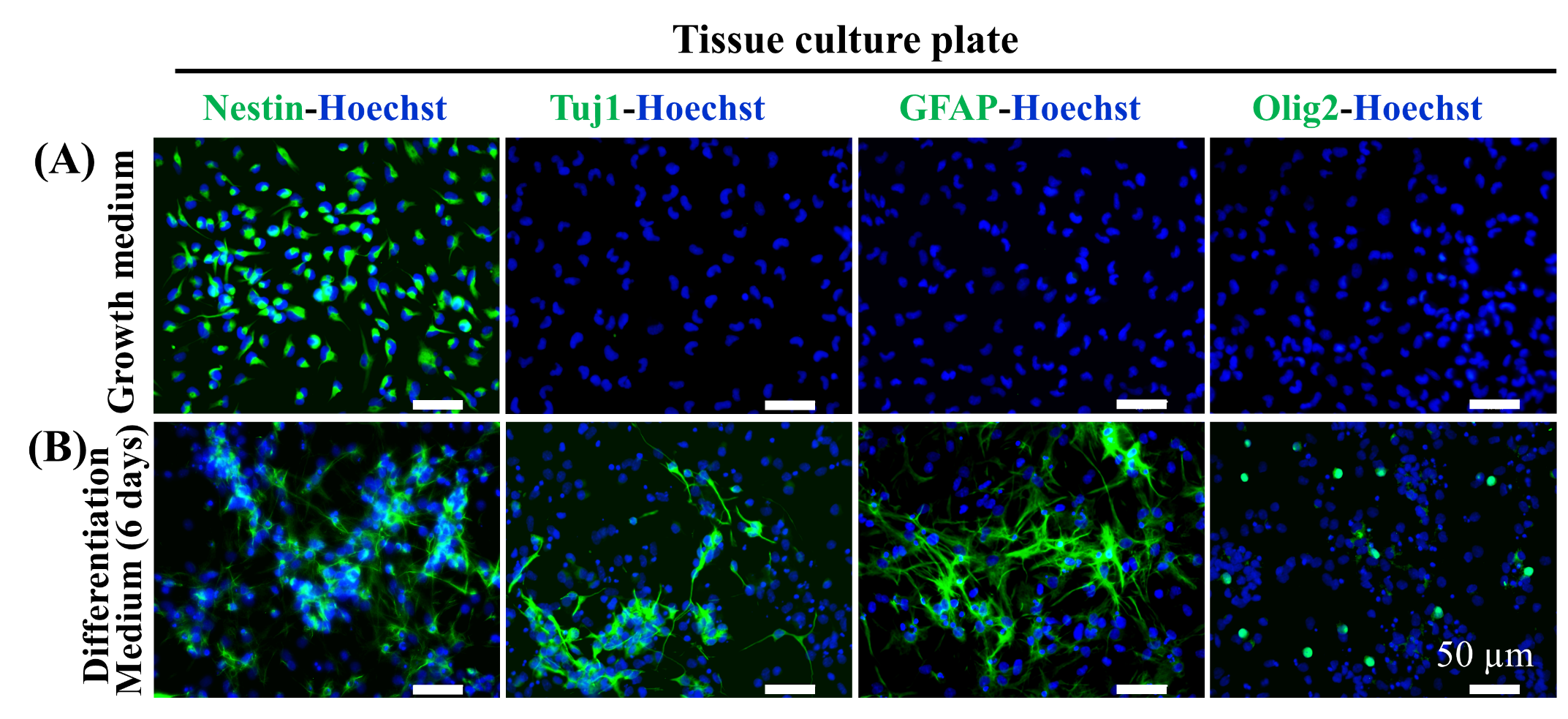


**Figure S5. Identification of primary cultured rat neural stem/progenitor cells (rNSPCs) and its differentiation on tissue culture plate.** (A) Neural stem cell maker (Nestin) staining for identification of rNSPCs under growth medium. Neural lineage-specific markers (Tuji, GFAP, and Olig2) were not stained from rNSPCs on a tissue culture plate (TCP). (B) Differentiation potential of rNSPCs was confirmed by fluorescence staining of neuronal (Tuj1), astrocyte (GFAP) and oligodendrocyte (Olig2) after 6 days’ differentiation on TCP under spontaneous differentiation medium in the absence of growth factors.


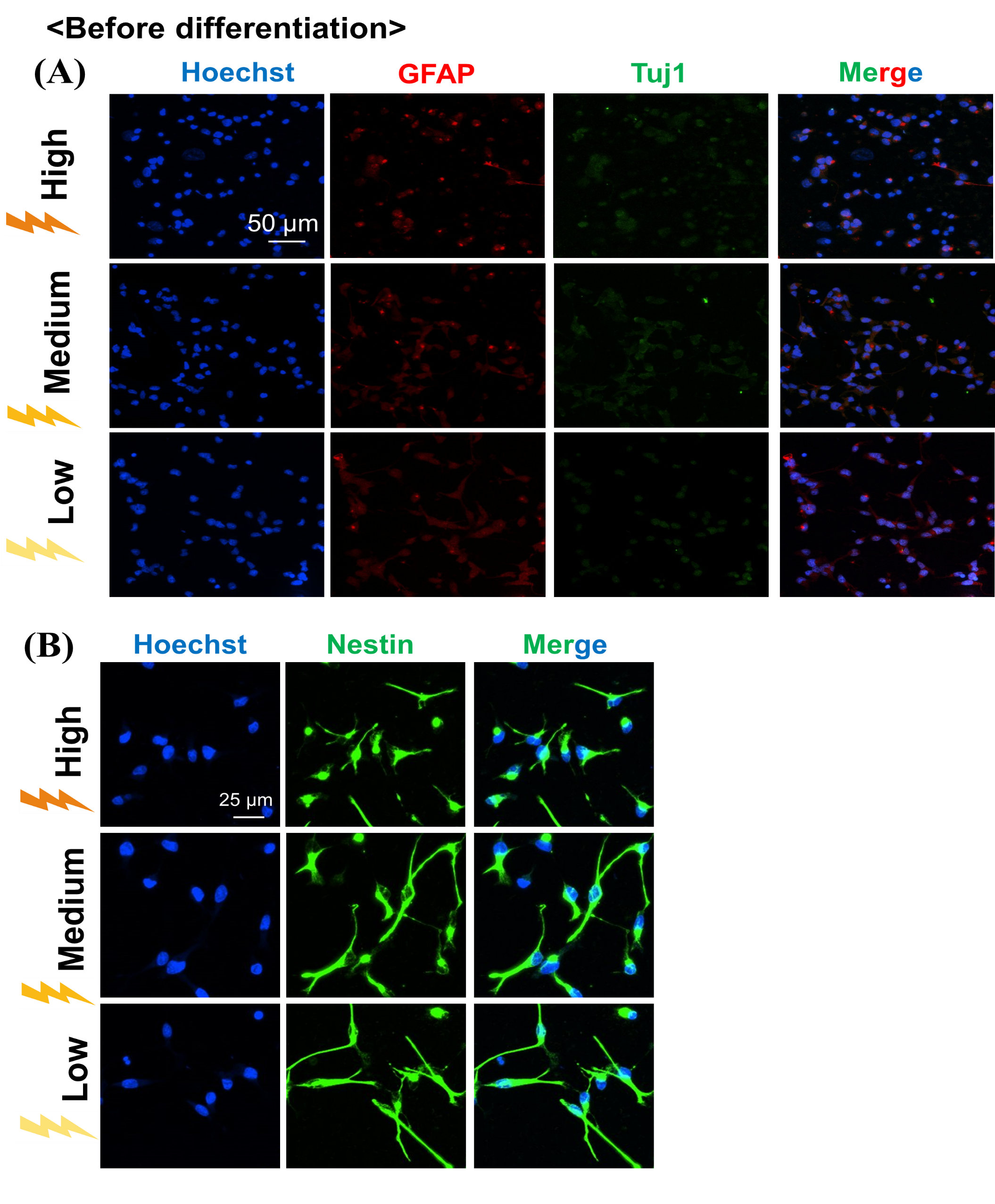


**Figure S6. Status of primary cultured rat neural stem/progenitor cells (rNSPCs) before differentiation on different conductivity.** (A) Neural lineage-specific markers (Tuji and GFAP) were not stained properly from rNSPCs before differentiation (at 24 hr seeding under growth medium). (B) Neural stem cell maker (Nestin) was well stained for identification of rNSPCs before differentiation.


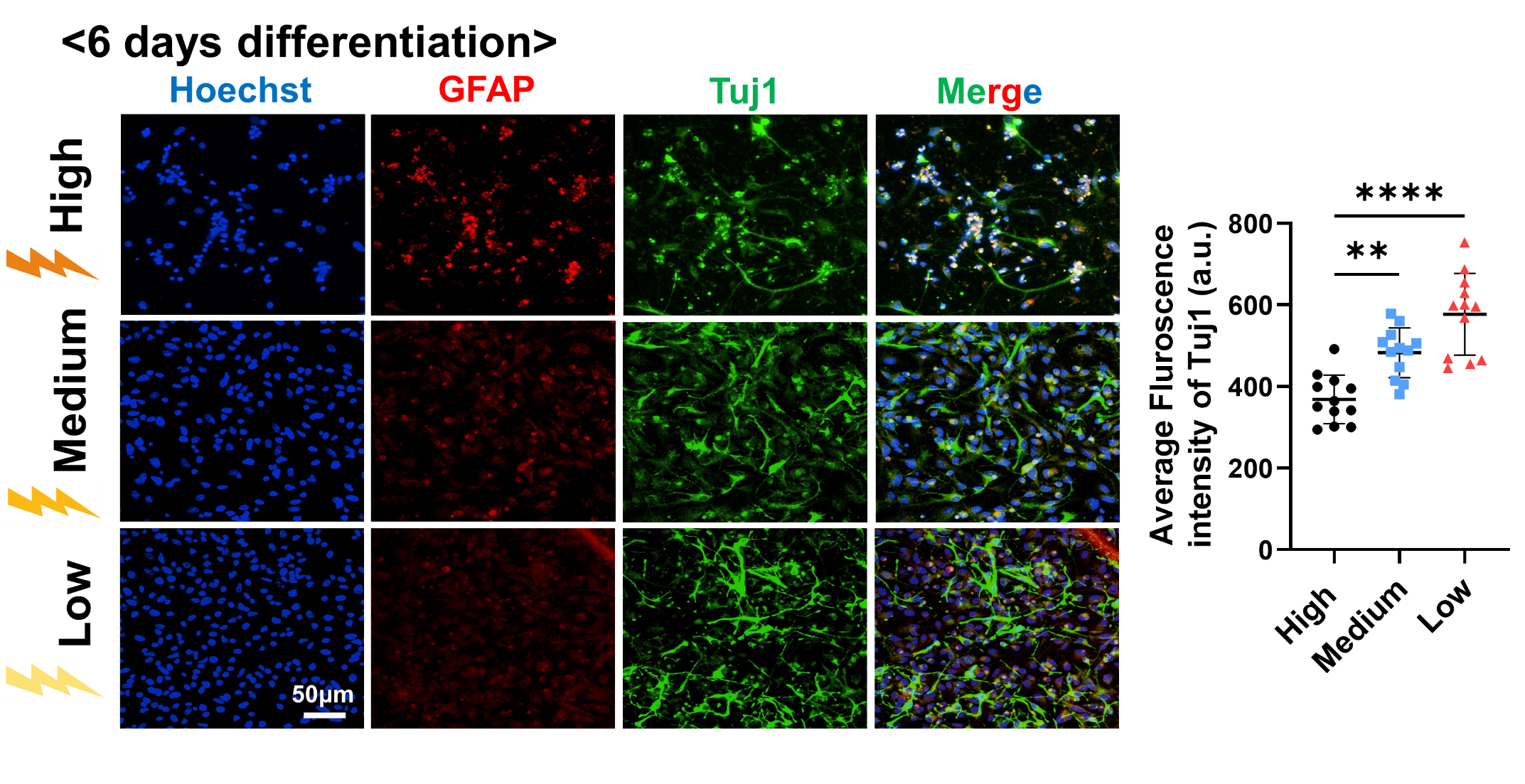


**Figure S7.** **Enhanced neural differentiation on neural tissue-like electrical conductivity (low and medium) is confirmed by GFAP and Tuj1 co-staining.** The neuronal differentiation of rat NSPC was examined by immunostaining of the neuronal maker Tuj1 after culture in the spontaneous differentiation conditions under growth factors withdrawal media for 6 days. Blue; nucleus (Hoechst staining), Red; astrocyte marker (GFAP), Green; early stage neuron marker (Tuj1). **P < 0.01 and ****P < 0.0001 between groups (ANOVA and tukey posthoc test after confirming normality and distribution symmetry by Shapiro-Wilk test at a level of 0.05).


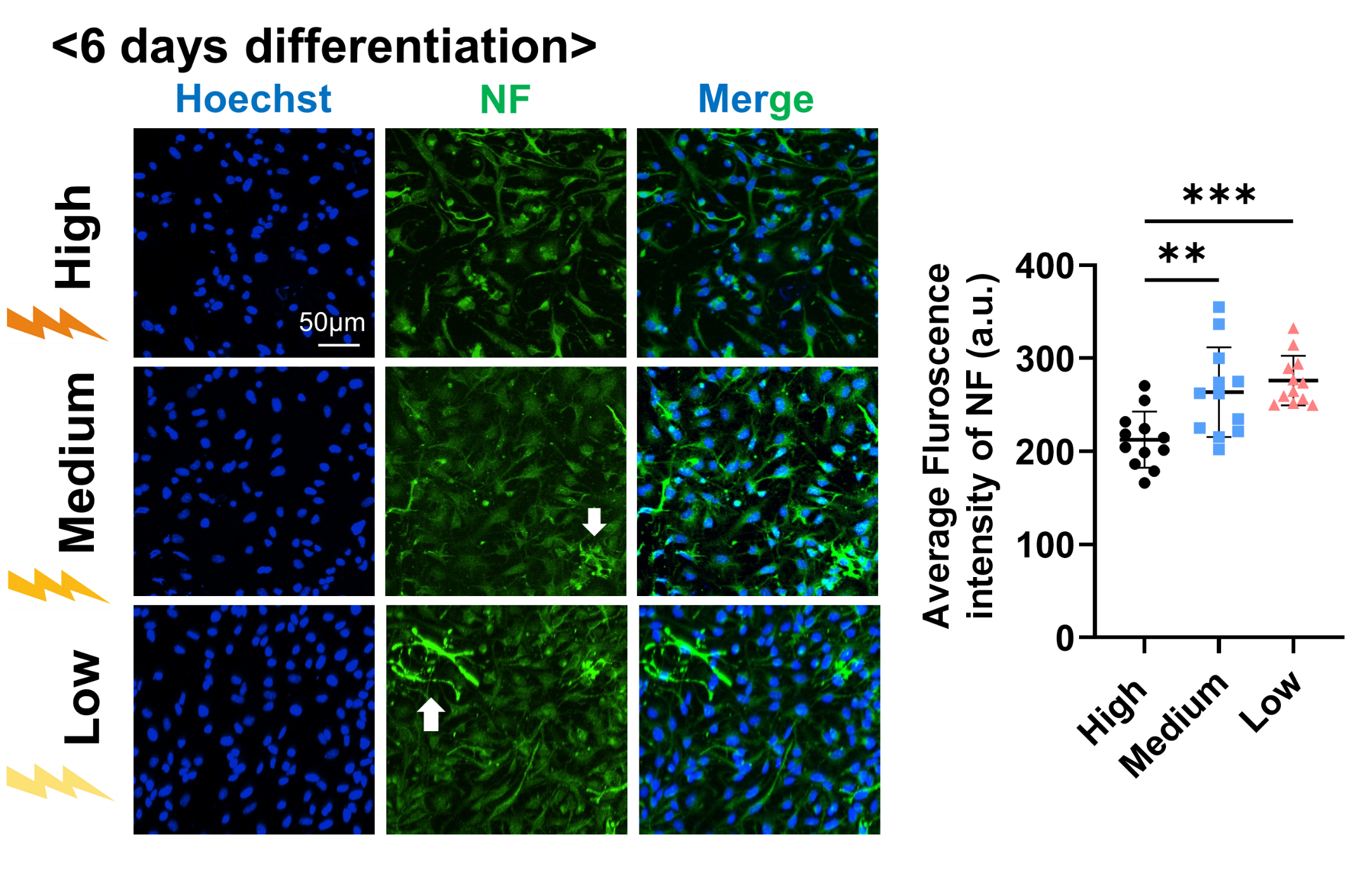


**Figure S8.** **Enhanced neural differentiation on neural tissue-like electrical conductivity (low and medium) is confirmed by neurofilament staining at day 6 of differentiation.** The neuronal differentiation of rat NSPC was examined by immunostaining of the pan-axonal neurofilament (NF) after culture in the spontaneous differentiation conditions under growth factors withdrawal media for 6 days. White arrows indicating strongly positive cells. Blue; nucleus (Hoechst staining), Green; pan-axonal neurofilament (NF, SIM312). **P < 0.01 and ***P < 0.001 between groups (ANOVA and tukey posthoc test after confirming normality and distribution symmetry by Shapiro-Wilk test at a level of 0.05).


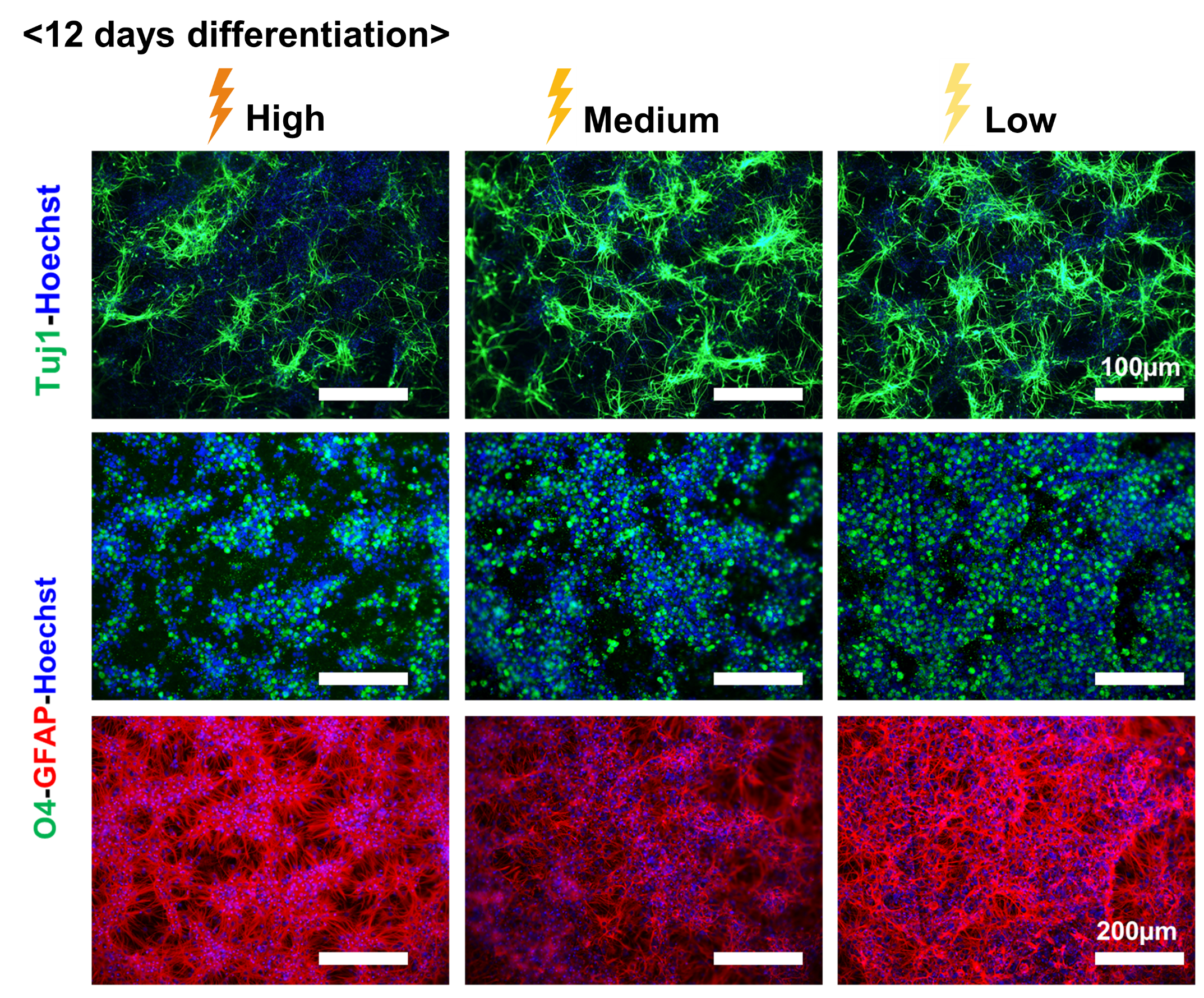


**Figure S9. Differentiation potential of rNSPCs cultured on different conductive carbon films at day 12 of differentiation**. TuJ1 stained cells with neurite growth were still well observed in ‘Medium’ and ‘Low’ electroconductivity substrate than ‘High’, while astrocyte marker, GFAP, stained cells were oppositely detected.


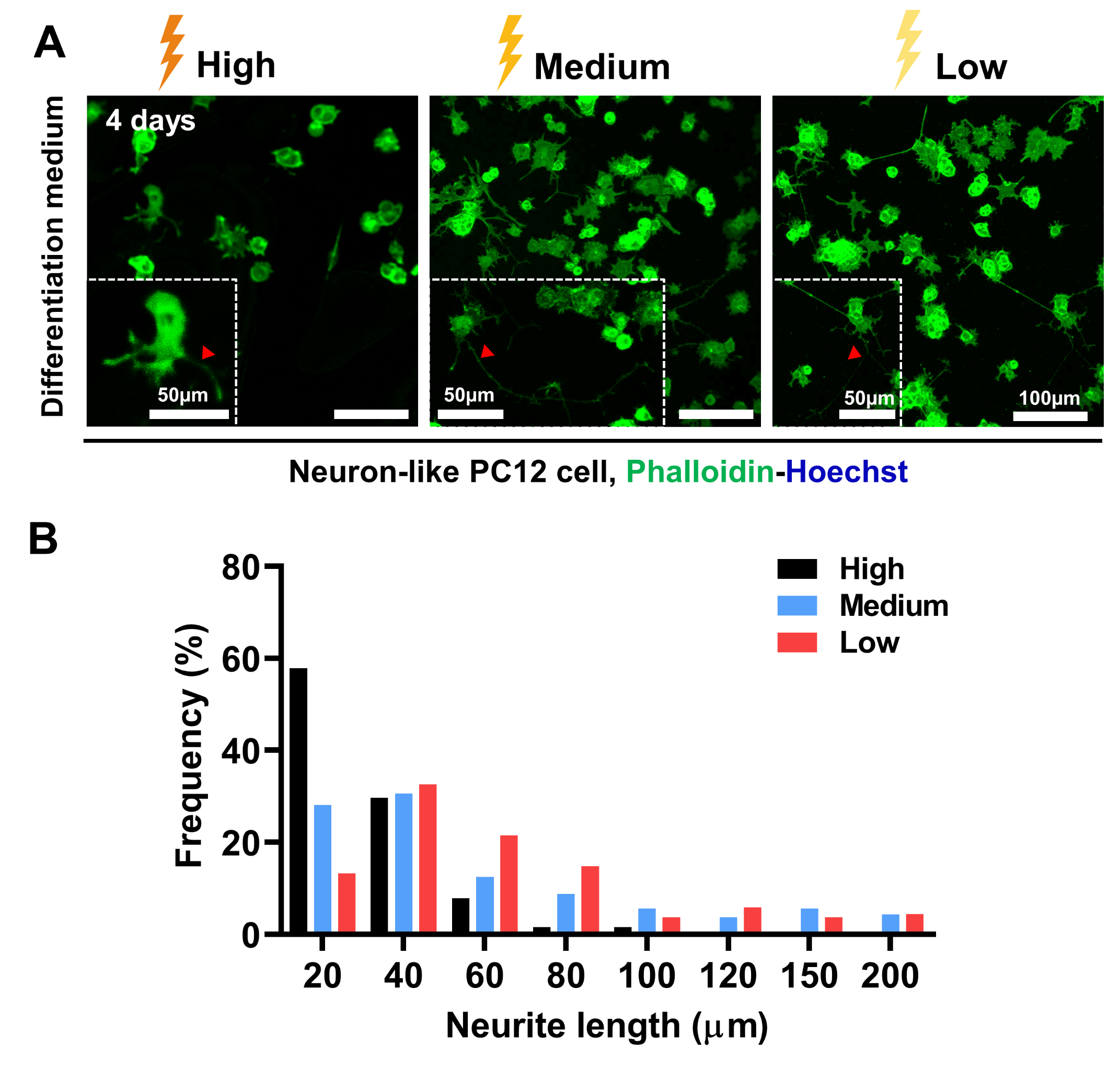


**Figure S10. Differentiation potential of PC12 cultured on different conductive carbon films at day 4.** NGF response behaviours were more dominant in the less conductive carbon film group. (**A**) When PC12 cells were cultured in a differentiation medium containing 100 ng/ml NGFs for 4 days, a neurite outgrowth was more observed in ‘Medium’ and ‘Low ‘conductivity. (**B**) Quantification analysis of NGF-response behaviours of PC12 cells showed enhanced average neurite length, notably elongated neurite, particularly exceeding 60, 80 or 120 µm.


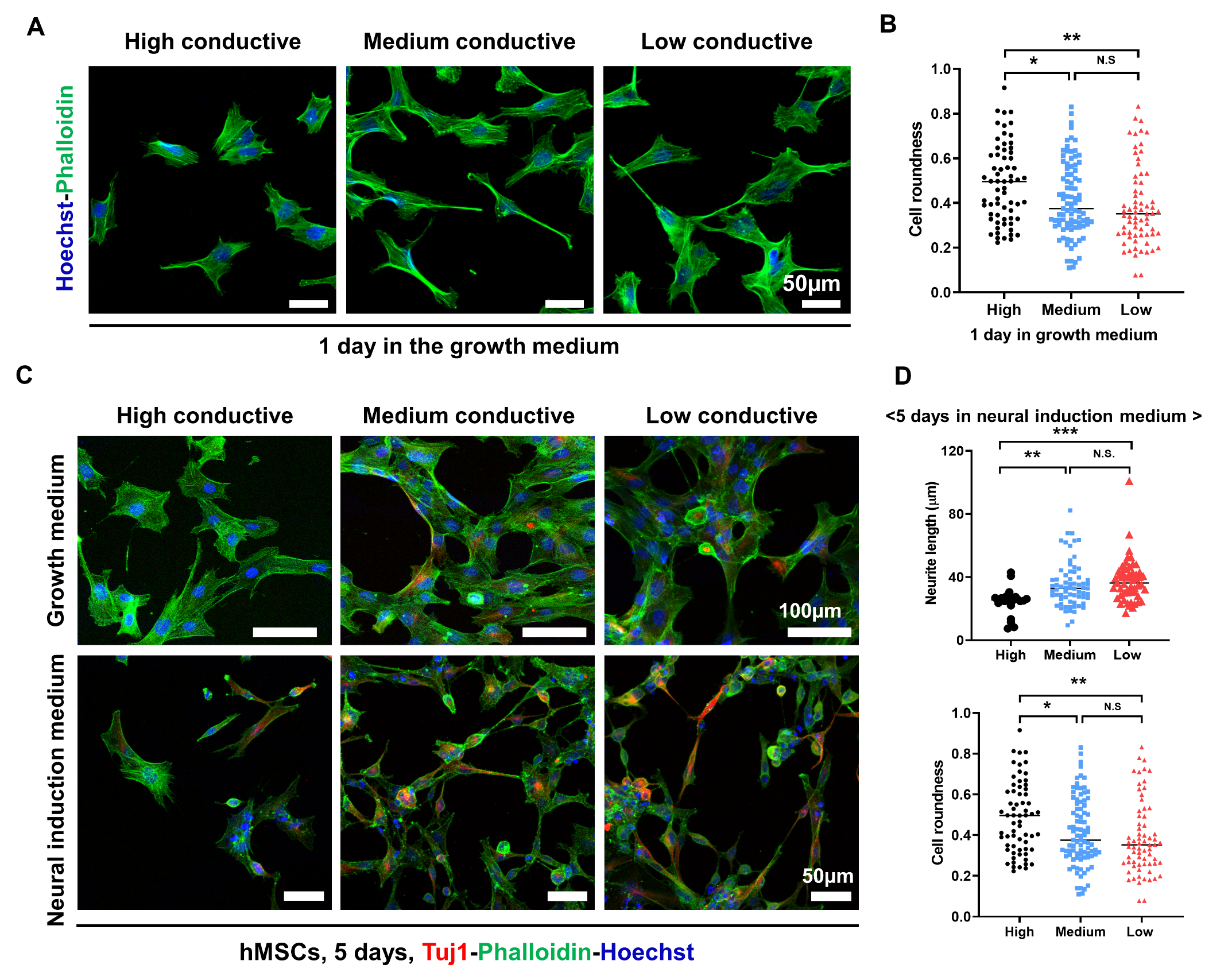


**Figure S11. Cellular response behaviours of hMSC cultured on the different electroconductive carbon films.** (**A&B**) 5,000 cells were seeded on carbon films (diameter 8 mm). 24 hours later, the cell roundness was further analyzed. The cell is elongated in ‘Medium’ and ‘Low’ conductivity. (**C**) After culture for 5 days in 2% FBS growth media or neural induction media, neuronal marker (TuJ1) is highly expressed n ‘Medium’ and ‘Low’ electroconductivity under growth or neural induction medium. (**D**) Neurite length and cell roundness at day 5 under neural induction medium. The neural induction media was composed of a mixture of DMEM/F12 and Neurobasal media [1:1 v/v] supplemented with 0.5% (v/v) N2, 1% (v/v) B27, 100 µM cAMP, and 20 ng/mL bFGF. ^*^*P* < 0.05, ^**^*P* < 0.01 and ^***^*P* < 0.001 (ANOVA and tukey posthoc test after confirming normality and distribution symmetry by Shapiro-Wilk test at a level of 0.05). N.S. indicated there was no significant difference between groups.


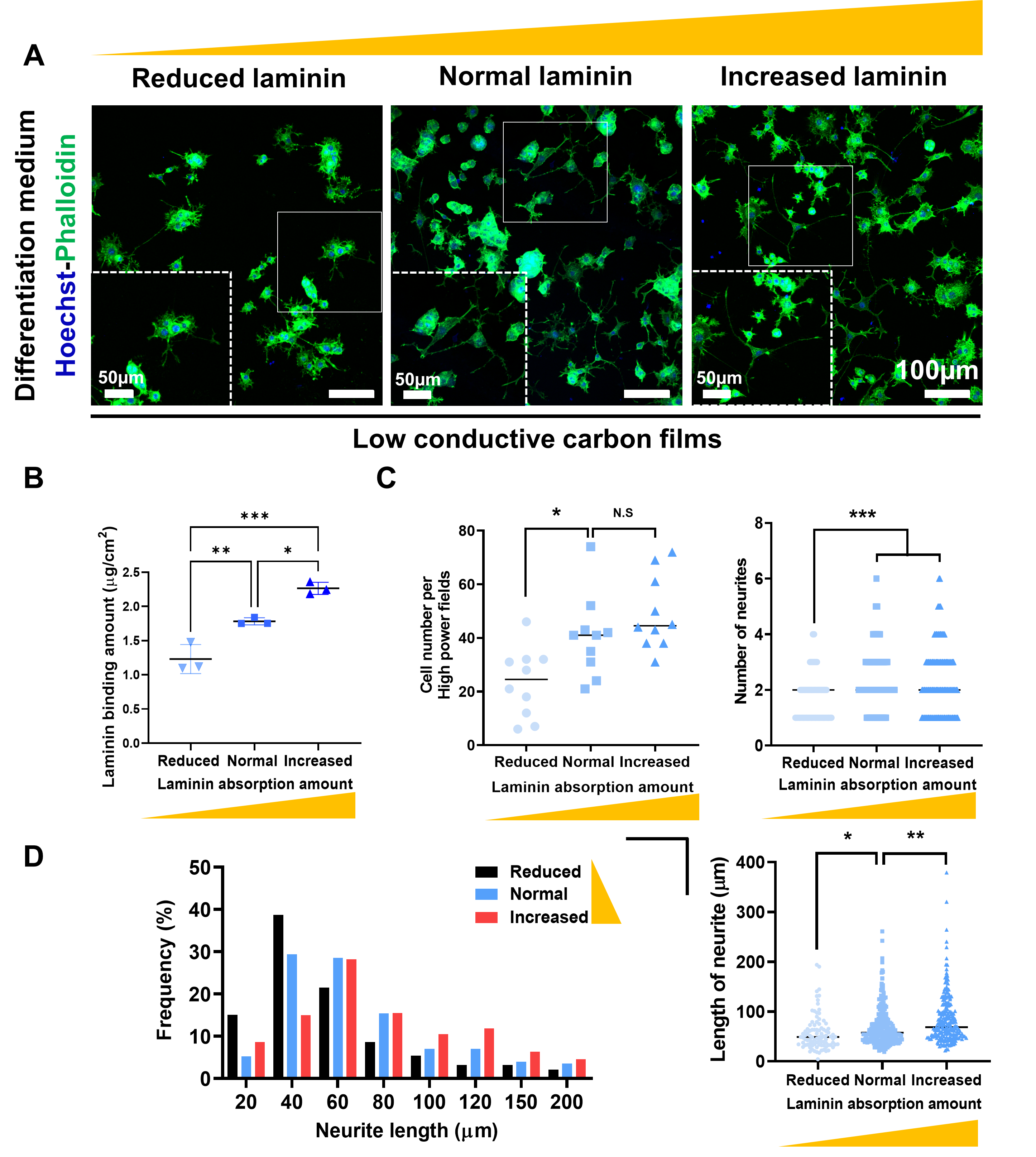


**Figure. S12. Neurogenic ECM, laminin, amount dependent neural cell response in ‘Low’ electroconductivity.** (**A**) Represent fluorescence images of PC12 cells cultured on the low conductive carbon film with different laminin coating amounts. The area within the rectangular white outline is magnified and presented in the inset delineated by the dotted white line for each image. NGFs response of PC12 was reduced on the low conductive carbon film group with lower laminin coating amount at day 4. (**B**) Different laminin coating amounts in low conductive carbon films. The normal condition is optimally matched amount among groups. (**C-D**) Quantification analysis for the adhesion cell number, cell morphology in terms of neurites numbers and length under NGFs (100 µg/ml) for 4 days. The neurite outgrowth response behaviour of PC12 cells was enhanced over the increasing amount of laminin protein in the low conductive carbon films. ^*^*P* < 0.05, ^**^*P* < 0.01 and ^***^*P* < 0.001 (ANOVA and tukey posthoc test after confirming normality and distribution symmetry by Shapiro-Wilk test at a level of 0.05). N.S. indicated there was no significant difference between groups.

**
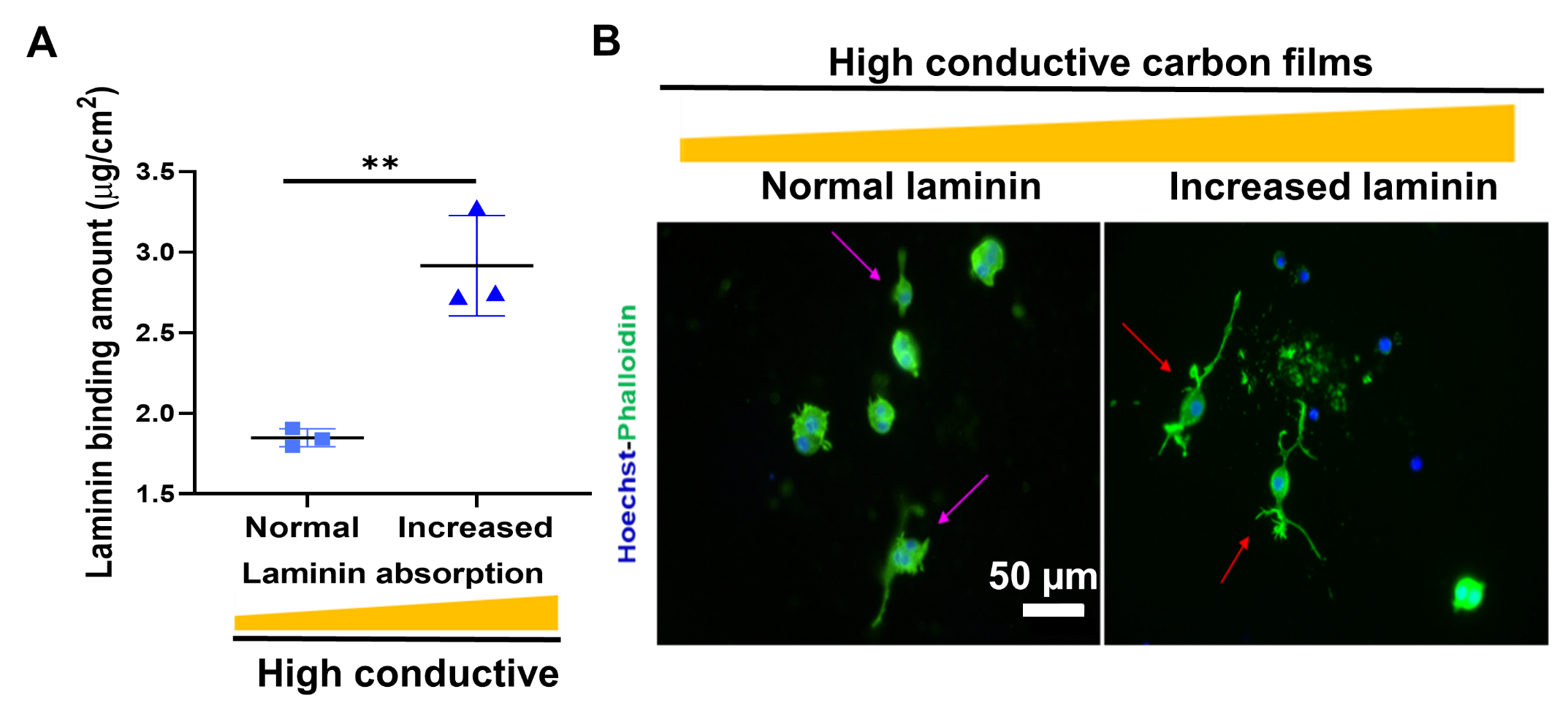
**

**Figure. S13. Neurogenic ECM, laminin, amount dependent neural cell response in ‘High’ electroconductivity. (A)** Increased laminin coating amount from normal condition (optimally matched amount among groups) on ‘High’ electroconductive substrate revealed (B) enhanced neurites outgrowth of PC12. When we increased the laminin coating amount in the High conductive carbon film group, the PC12 cells showed an increased neurite outgrowth response under NGFs. ^**^*P* < 0.01 (t-test after confirming normality and distribution symmetry by Shapiro-Wilk test at a level of 0.05).


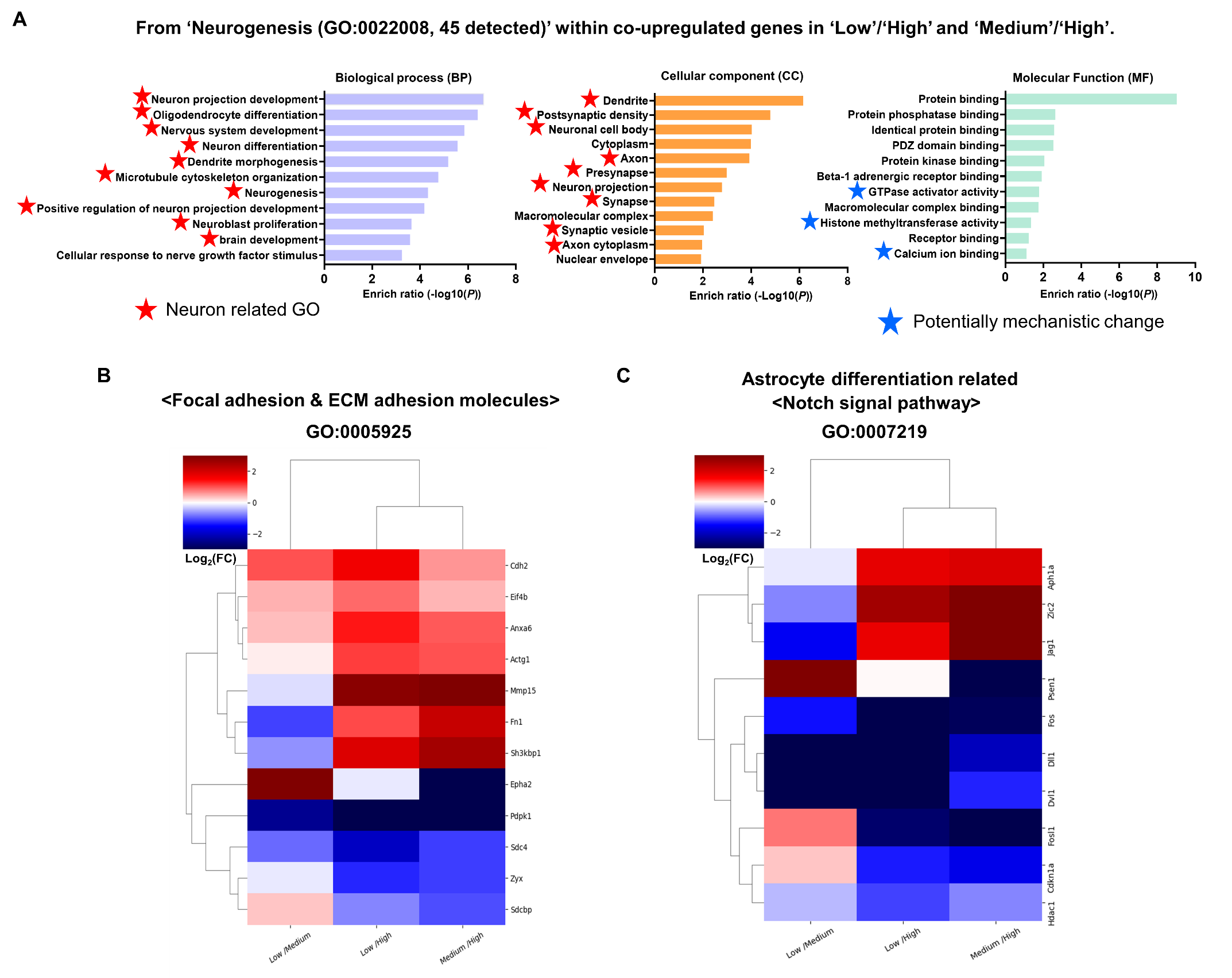


**Fig. S14. Neurogenesis-related critical signal pathways.** (A). Go analysis for neurogenesis-related DEGs. The red star indicated the "neuron-related process". Blue star indicated the "potential related process". (B). A heatmap for the "Focal adhesion & ECM adhesion molecules". (C). A heatmap for the "Notch signal pathway".


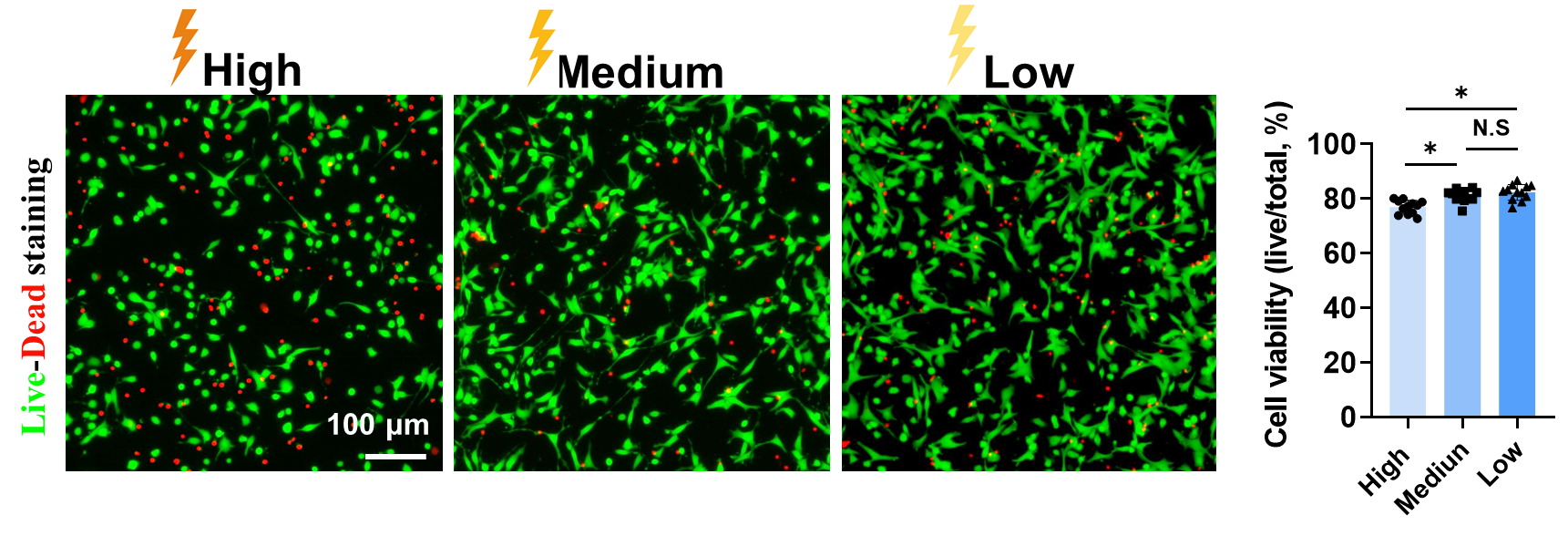


**Figure.S15. Live and dead images after 24 cultures on different conductivity.** Dead cells are slightly more detected in High conductivity than others. ^*^*P* < 0.05 (ANOVA and tukey posthoc test after confirming normality and distribution symmetry by Shapiro-Wilk test at a level of 0.05). N.S indicated there was no significant difference between groups.


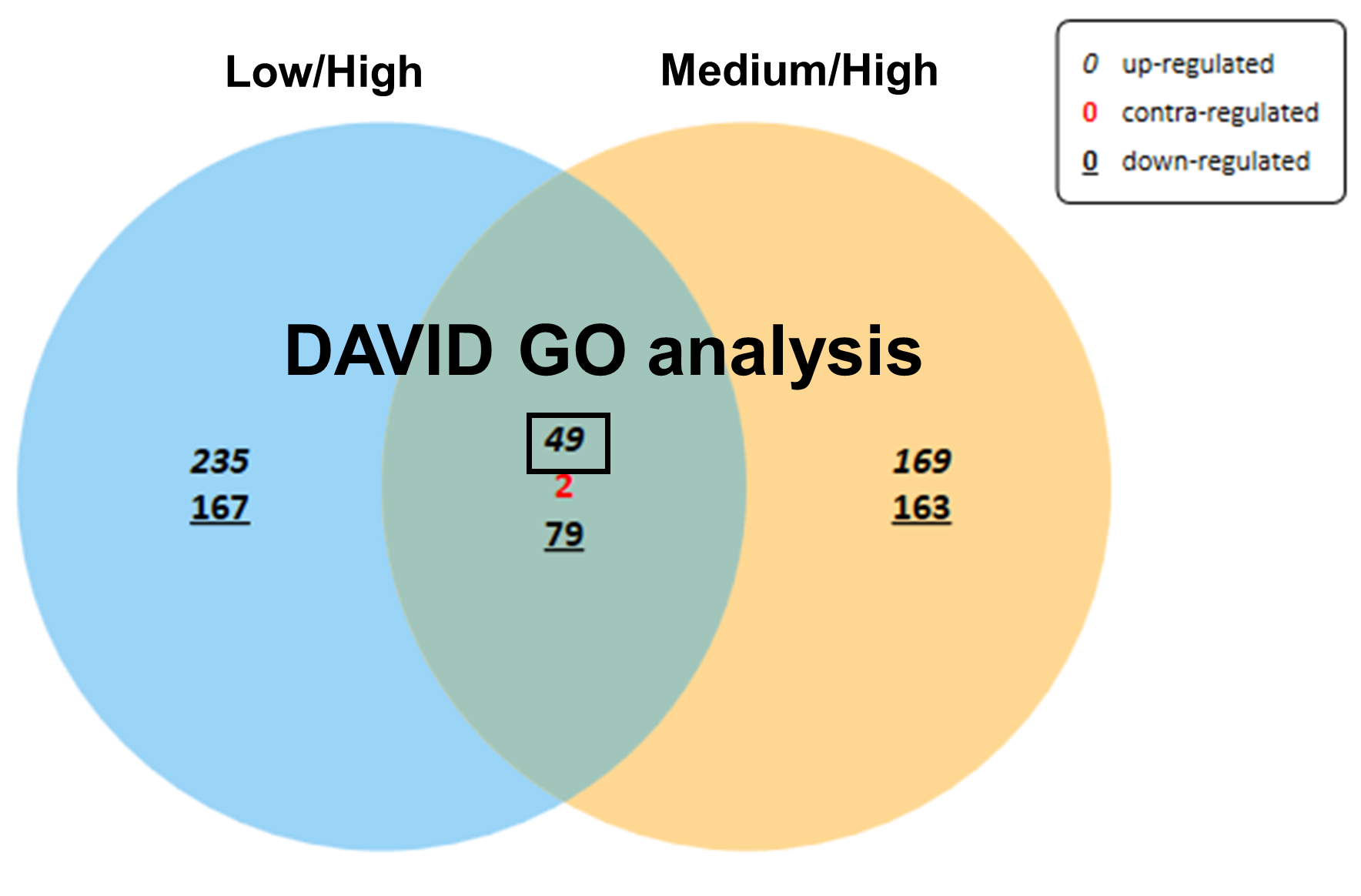


**Figure S16. Venn diagram figure illustrated the interested DEGs for further David GO analysis of co-upregulated genes in neural tissue like electroconductivity**. GO analysis for the co-upregulated DEGs in the ‘Medium’ and ‘Low’ conductive carbon film groups was performed compared to ‘High’ (**Fig. 4B&C**).


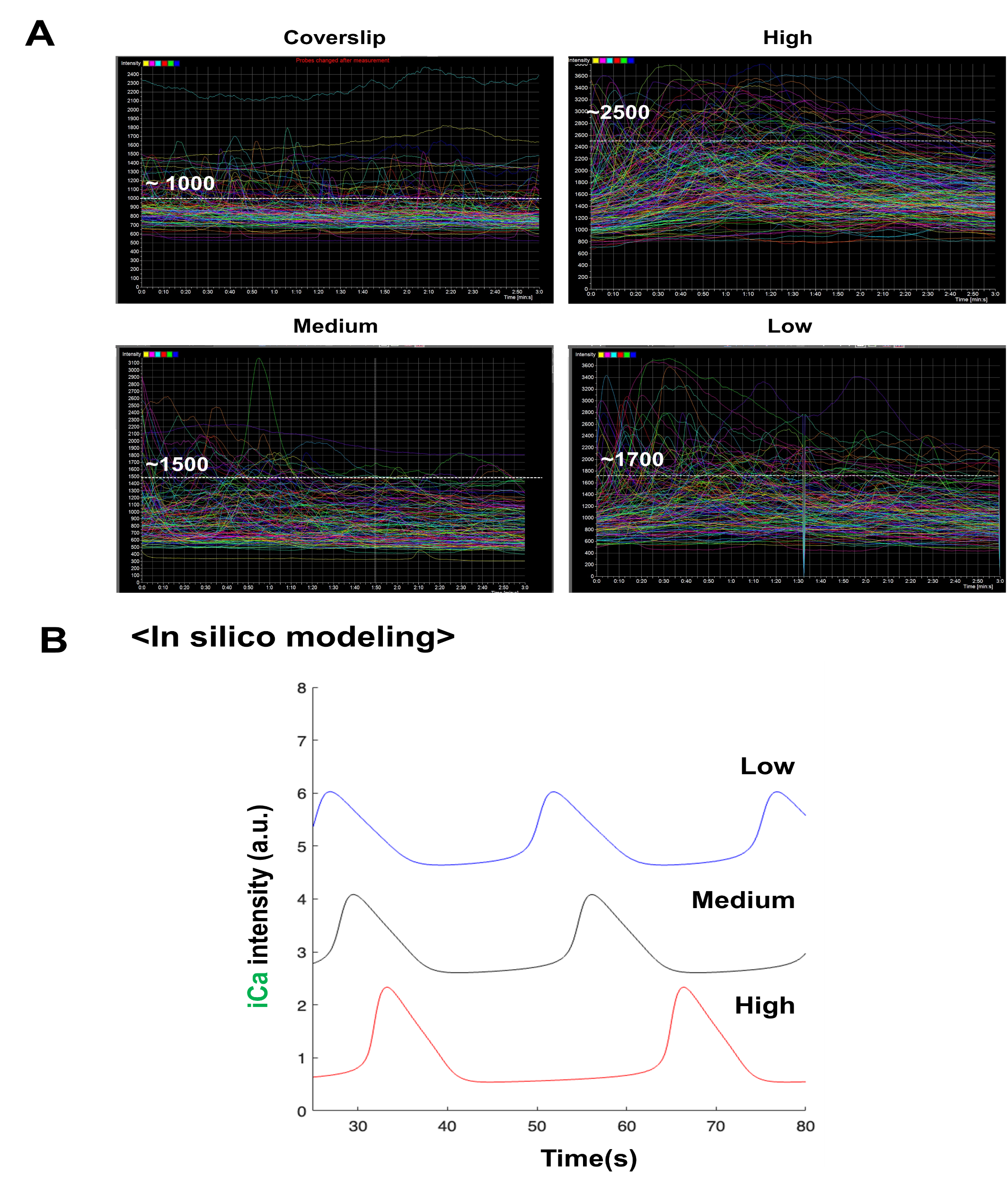


**Figure S17. Overall calcium oscillations analysis and in silico modeling of rNSPCs cultured on different conductive carbon films.** (A) Overall view of calcium oscillations from a large number of cells. The average calcium intensity value was shown in each panel. Cells on a coverslip were used as control. (B) In silico modeling using different conductivity. It revealed high intracellular calcium in ‘High’ conductivity.


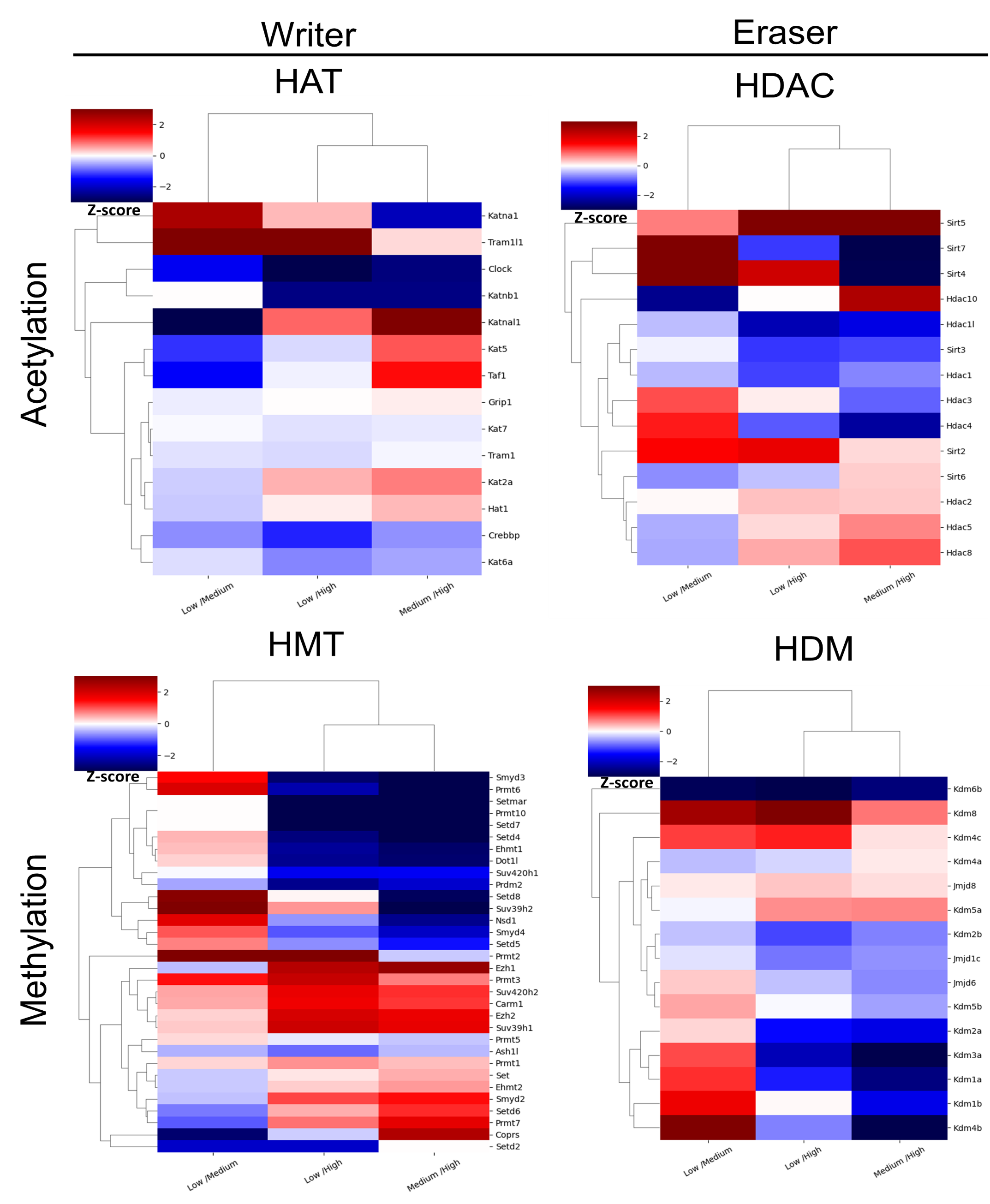


**Figure S18. The gene expression level of histone acetylation and methylation modifiers by RNA-seq.**


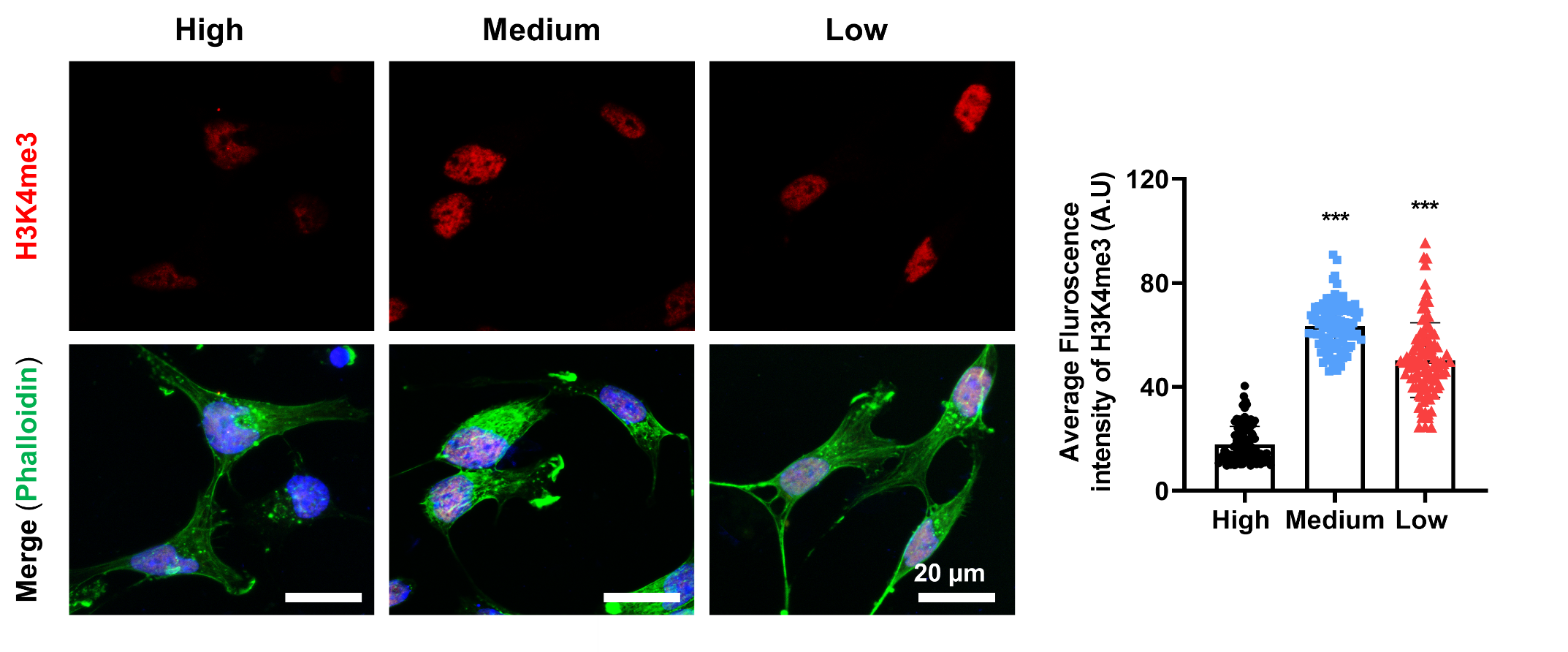


**Figure S19. Euchromatic H3K4me3 expression of rNSPCs cultured on different conductive carbon films.** H3K4me3 expression was highly detected in ‘medium’ and ‘low’ than ‘high’. H3K4me3 is enriched at transcription start sites of active genes, promoting transcription. ^***^*P* < 0.001 compared to High (ANOVA and tukey posthoc after confirming normality and distribution symmetry by Shapiro-Wilk test at a level of 0.05).


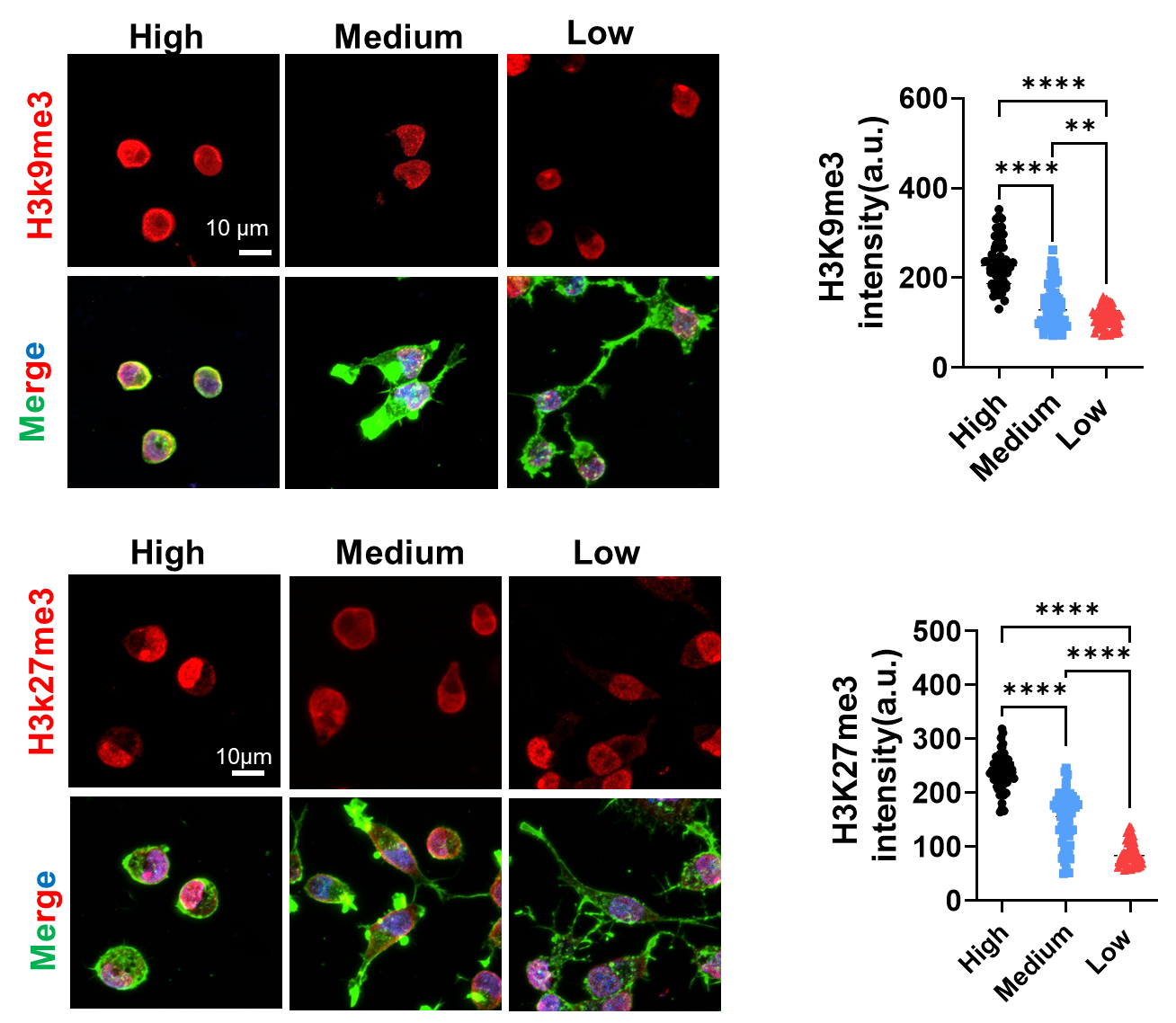


**Figure S20. Heterochromatic H3K9me3 and H3K27me3 expression of rNSPCs cultured on different conductive carbon films.** H3K9me3 and H3K27me3 expression were highly detected in ‘high’ than ‘medium’ and ‘low’. High expression of H3K9me3 and H3K27me3 indicates downregulation of nearby genes via the formation of heterochromatic regions. ^***^*P* < 0.001 compared to High (ANOVA and tukey posthoc after confirming normality and distribution symmetry by Shapiro-Wilk test at a level of 0.05).


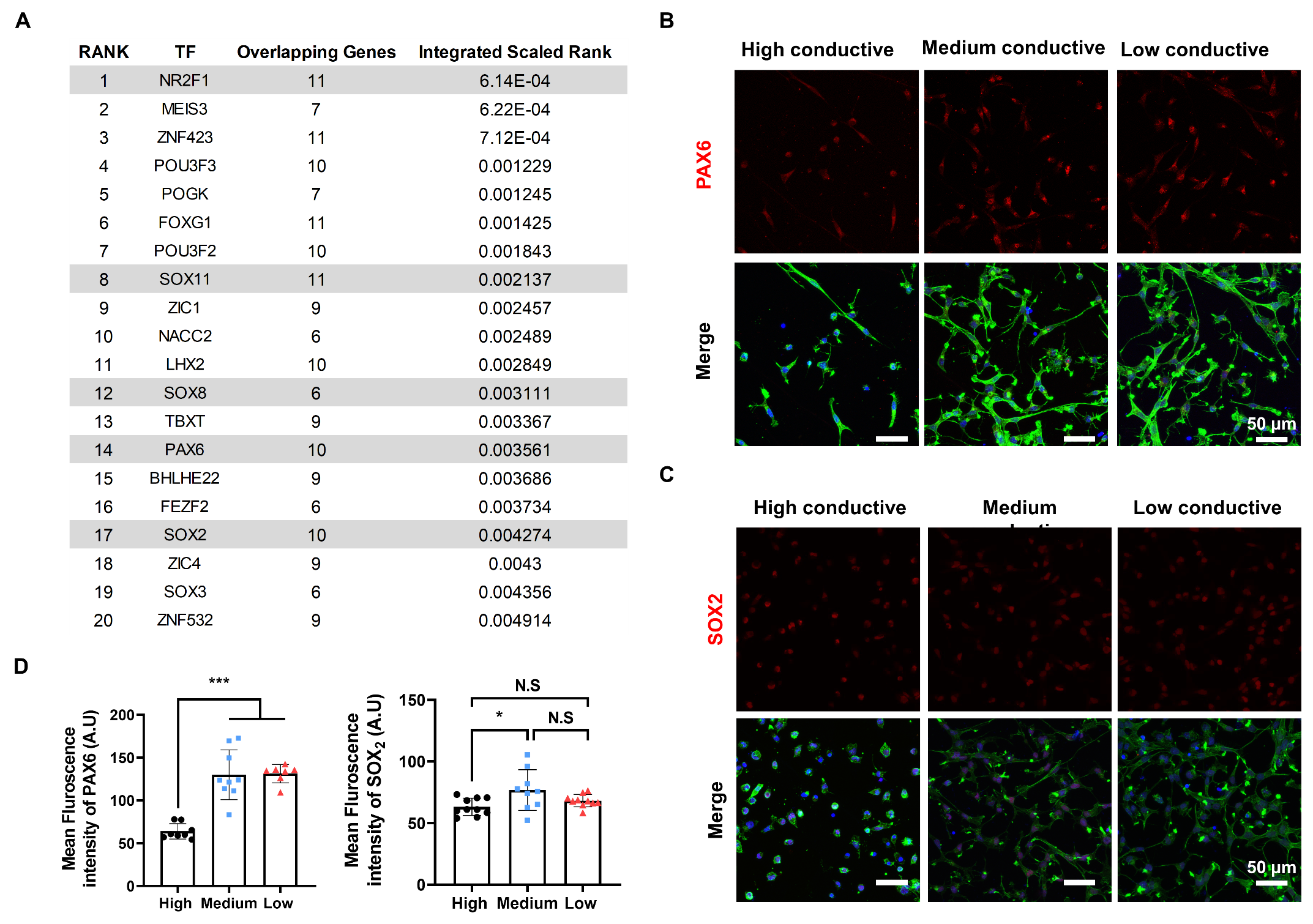


**Figure S21. Potential transcription factors mediating the different differentiation behaviours of rNSPCs cultured on different conductive carbon films.**  (**A**). Potential transcription factors involved in this study by using the ChIP-X Enrichment Analysis Version 3 (ChEA3). The gene set was collected with the condition "Neurogenesis" from both up-regulated DEGs in Medium or Low conductive group (over 1.5-fold change and Normalization 2). (**B-C**). SOX2 and PAX6 expression for rNSPCs cultured on the different conductive carbon films. (**D**) Quantification analysis for the SOX2 and PAX6 expression. ^*^*P* < 0.05 and ^***^*P* < 0.001 (ANOVA and tukey posthoc after confirming normality and distribution symmetry by Shapiro-Wilk test at a level of 0.05). N.S. indicated there was no significant difference between groups.

**
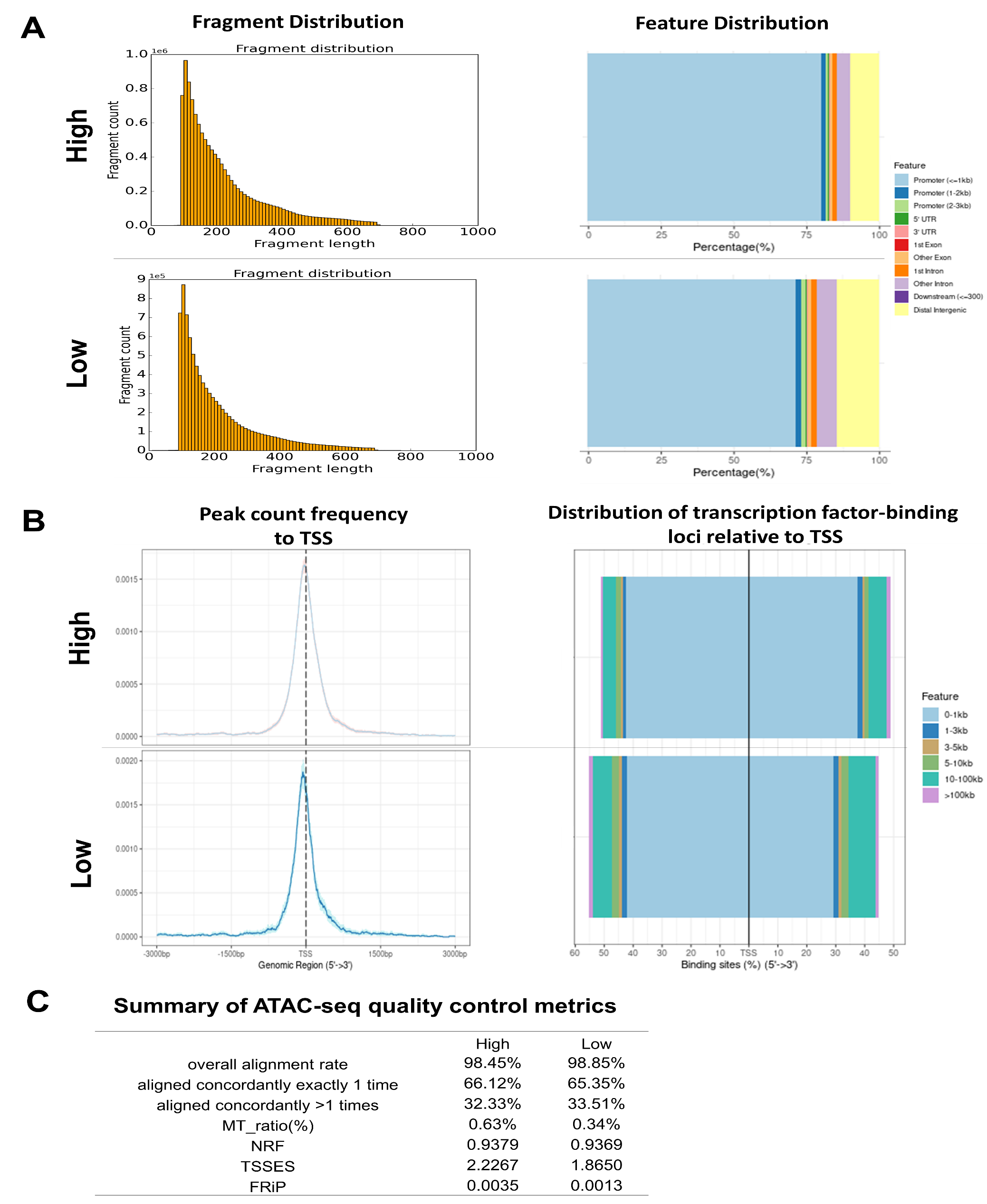
**

**Figure S22. Overview of assay for transposase-accessible chromatin using sequencing (ATAC-seq) data sets.** (A) Fragment and genome wide distribution. (B) Peak count frequency and distribution of transcription factor-binding loci relative to transcription starting site. (C) ATAC-seq quality control metrics.


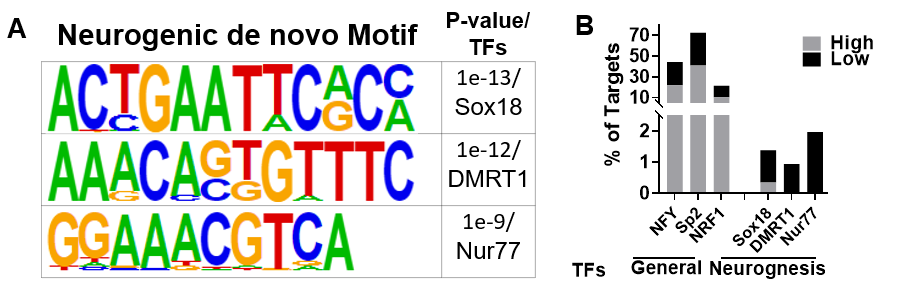


**Figure S23. Representative Homer de novo TF binding motifs.** (A) Hit neurogenic de novo motifs in low conductivity with p-value. (B) Percentage of target sequences containing de novo TF binding motifs, categorized into general and neurogenesis-specific transcription factors, revealing differential regulatory element enrichment to neurogenesis in response to physiologic substrate conductivity (low).

**References**

[1] Y.-M. Li, K. D. Patel, Y.-K. Han, S.-M. Hong, Y.-X. Meng, H.-H. Lee, J. H. Park, J. C. Knowles, J. K. Hyun, J.-H. Lee, H.-W. Kim, *Chemical Engineering Journal* **2023**, *466*, 143125.

[2] J. Landers, J. T. Turner, G. Heden, A. L. Carlson, N. K. Bennett, P. V. Moghe, A. V. Neimark, *Adv Healthc Mater* **2014**, *3*, 1745.

[3] H.-S. Ahn, J.-Y. Hwang, M. S. Kim, J.-Y. Lee, J.-W. Kim, H.-S. Kim, U. S. Shin, J. C. Knowles, H.-W. Kim, J. K. Hyun, *Acta Biomaterialia* **2015**, *13*, 324.

[4] A. K. Panda, R. K, A. Gebrekrstos, S. Bose, Y. S. Markandeya, B. Mehta, B. Basu, *ACS Appl. Mater. Interfaces* **2021**, *13*, 164.

[5] X. Hu, X. Wang, Y. Xu, L. Li, J. Liu, Y. He, Y. Zou, L. Yu, X. Qiu, J. Guo, *Advanced Healthcare Materials* **2020**, *9*, 1901570.

[6] J. Zhou, X. Yang, W. Liu, C. Wang, Y. Shen, F. Zhang, H. Zhu, H. Sun, J. Chen, J. Lam, A. G. Mikos, C. Wang, *Theranostics* **2018**, *8*, 3317.

[7] M. Mantecón-Oria, O. Tapia, M. Lafarga, M. T. Berciano, J. M. Munuera, S. Villar-Rodil, J. I. Paredes, M. J. Rivero, N. Diban, A. Urtiaga, *Sci Rep* **2022**, *12*, 13408.

[8] X. Yao, L. Zhan, Z. Yan, J. Li, L. Kong, X. Wang, H. Xiao, H. Jiang, C. Huang, Y. Ouyang, Y. Qian, C. Fan, *Bioactive Materials* **2023**, *20*, 319.

[9] Y. Qian, J. Song, X. Zhao, W. Chen, Y. Ouyang, W. Yuan, C. Fan, *Advanced Science* **2018**, *5*, 1700499.

[10] C. Dong, F. Qiao, W. Hou, L. Yang, Y. Lv, *Applied Materials Today* **2020**, *21*, 100870.

[11] J. Wang, Y. Cheng, L. Chen, T. Zhu, K. Ye, C. Jia, H. Wang, M. Zhu, C. Fan, X. Mo, *Acta Biomaterialia* **2019**, *84*, 98.

[12] G. Thrivikraman, G. Madras, B. Basu, *Biomaterials* **2014**, *35*, 6219.

[13] Y.-H. Zhao, C.-M. Niu, J.-Q. Shi, Y.-Y. Wang, Y.-M. Yang, H.-B. Wang, *Neural Regen Res* **2018**, *13*, 1455.

[14] M. Patel, J. H. Min, M.-H. Hong, H.-J. Lee, S. Kang, S. Yi, W.-G. Koh, *Biomed. Mater.* **2020**, *15*, 045007.

[15] M. Rahimzadegan, Q. Mohammadi, M. Shafieian, O. Sabzevari, Z. Hassannejad, *Biomaterials Advances* **2022**, *134*, 112634.

[16] S. Vijayavenkataraman, N. Vialli, J. Y. H. Fuh, W. F. Lu, *International Journal of Bioprinting* **Invalid date**, *5*, 0.

[17] E. A. Kiyotake, E. E. Thomas, H. B. Homburg, C. K. Milton, A. D. Smitherman, N. D. Donahue, K.-M. Fung, S. Wilhelm, M. D. Martin, M. S. Detamore, *Journal of Biomedical Materials Research Part A* **2022**, *110*, 365.

[18] F. R. Boroojeni, S. Mashayekhan, H.-A. Abbaszadeh, M. Ansarizadeh, M.-S. Khoramgah, V. R. Movaghar, *IJN* **2020**, *15*, 3903.

[19] C. Wu, S. Chen, T. Zhou, K. Wu, Z. Qiao, Y. Zhang, N. Xin, X. Liu, D. Wei, J. Sun, H. Luo, L. Zhou, H. Fan, *ACS Appl. Mater. Interfaces* **2021**, *13*, 52346.

[20] N. Pelot, B. Thio, W. Grill, *Frontiers in Computational Neuroscience* **2018**, *12*.

[21] Influence of head models on EEG simulations and inverse source localizations | BioMedical Engineering OnLine | Full Text, .

[22] A. Hirata, Y. Takano, Y. Kamimura, O. Fujiwara, *Physics in medicine and biology* **2010**, *55*, N243.

[23] B. Howell, S. Lad, W. Grill, *PloS one* **2014**, *9*, e114938.
